# Hydrostatic pressure promotes endothelial tube formation through aquaporin 1 and Ras-ERK signaling

**DOI:** 10.1038/s42003-020-0881-9

**Published:** 2020-04-02

**Authors:** Daisuke Yoshino, Kenichi Funamoto, Kakeru Sato, Kenry, Masaaki Sato, Chwee Teck Lim

**Affiliations:** 1 Frontier Research Institute for Interdisciplinary Sciences, Tohoku University, 6-3 Aramaki-Aoba, Aoba-ku, Sendai, 980-8578 Japan; 2 Mechanobiology Institute, National University of Singapore, #10-01 T-Lab, 5A Engineering Drive 1, Singapore, 117411 Singapore; 3 Institute of Fluid Science, Tohoku University, 2-1-1 Katahira, Aoba-ku, Sendai, 980-8577 Japan; 4 Institute of Engineering, Tokyo University of Agriculture and Technology, 2-24-16 Naka-cho, Koganei, Tokyo, 184-8588 Japan; 5 Graduate School of Engineering, Tohoku University, 6-6-01 Aramaki-Aoba, Aoba-ku, Sendai, 980-8579 Japan; 6 Department of Biomedical Engineering, National University of Singapore, 4 Engineering Drive 3, Singapore, 117583 Singapore; 7 Institute for Health Innovation and Technology (iHealthtech), National University of Singapore, #14-01 MD6, 14 Medical Drive, Singapore, 117599 Singapore; 8 Present Address: Tokyo Gas Co., Ltd., 1-5-20 Kaigan, Minato-ku, Tokyo, 105-8527 Japan

**Keywords:** Biophysics, Angiogenesis, Cell signalling

## Abstract

Vascular tubulogenesis is tightly linked with physiological and pathological events in the living body. Endothelial cells (ECs), which are constantly exposed to hemodynamic forces, play a key role in tubulogenesis. Hydrostatic pressure in particular has been shown to elicit biophysical and biochemical responses leading to EC-mediated tubulogenesis. However, the relationship between tubulogenesis and hydrostatic pressure remains to be elucidated. Here, we propose a specific mechanism through which hydrostatic pressure promotes tubulogenesis. We show that pressure exposure transiently activates the Ras/extracellular signal-regulated kinase (ERK) pathway in ECs, inducing endothelial tubulogenic responses. Water efflux through aquaporin 1 and activation of protein kinase C via specific G protein–coupled receptors are essential to the pressure-induced transient activation of the Ras/ERK pathway. Our approach could provide a basis for elucidating the mechanopathology of tubulogenesis-related diseases and the development of mechanotherapies for improving human health.

## Abstract (web summary)

Yoshino et al. investigate the mechanism by which exposure to pressure promotes endothelial cells to form tubes and find that Aquaporin-mediated water efflux activates the Ras-ERK pathway via PKC and GPCR activation. These findings may be relevant to understand how blood pressure affects vascular tubulogenesis.

## Introduction

Blood vessels play important roles in the maintenance of homeostasis (maintenance of a normal physiologic state) because they are essential for supplying oxygen and nutrients to every part of the body. Pathologically, blood vessels can also play an important role in the breakdown of homeostasis such as delivering nourishment to tumors, as is the case for certain cancers^1^. Hence, the formation of blood vessel/capillary networks is tightly linked with both normal physiology and pathology. Vascular tubulogenesis, which is central to the process through which these networks are formed, thus supports developmental processes^2^ as well as physiologic or pathologic growth of tissues^1,3,4^. This tubulogenic process is typically regulated by various responses of vascular endothelial cells (ECs), including adhesion, migration, and proliferation^5,6^. These responses, in turn, are elicited by hemodynamic stimuli generated in vivo in the circulatory system, such as cyclic stretching of tissues^7^, fluid shear stress^8,9^, and hydrostatic pressure^10^. Hydrostatic pressure has recently attracted considerable attention as a key stimulus that enhances tubulogenesis mediated by ECs because hydrostatic pressure is a stimulus that can be easily regulated through exercise^11,12^ and blood pressure medication. Depending on the local conditions, magnitude, and mode by which it is exerted, hydrostatic pressure can enhance the three-dimensional migration, cell cycle progression, endothelial proliferation, sprouting angiogenesis, and apoptosis of ECs^13–16^. Nevertheless, knowledge regarding how cells respond to hydrostatic pressure remains limited in terms of the mechanism through which pressure promotes angiogenesis during the maintenance and breakdown of homeostasis.

Here, we show the mechanism through which hydrostatic pressure promotes endothelial tubulogenesis. We found that pressure-induced transient activation of the Ras/extracellular signal–regulated kinase (ERK) pathway plays a crucial role in the promotion of tubulogenesis. We also confirmed that pressure-induced transient activation of the Ras/ERK pathway requires water efflux through aquaporin 1 (AQP1) and activation of protein kinase C (PKC) via a specific G protein–coupled receptor (GPCR).

## Results

### Hydrostatic pressure promotes endothelial tube formation

**Fig. 1:**
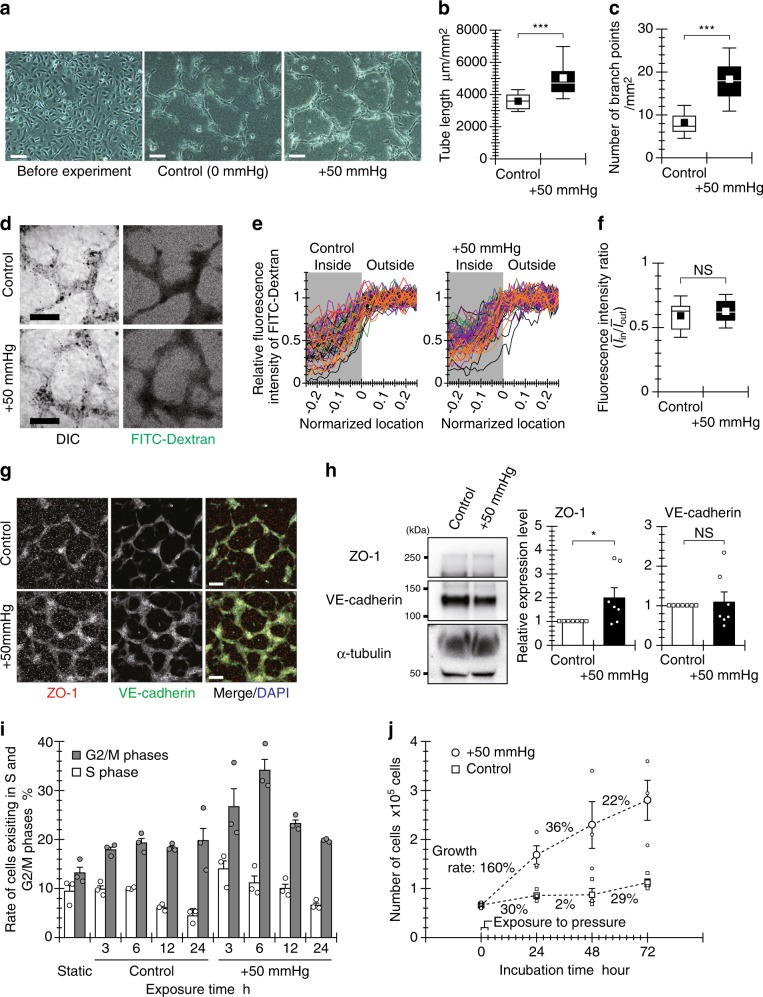
Hydrostatic pressure promotes endothelial tubulogenesis. **a** Endothelial tube formation under the pressure condition. ECs were embedded within a collagen gel sandwich and exposed to pressure for 3 h or incubated in the control condition for 13 h. Scale bars, 100 µm. **b** Quantified total length of tube-like structure and **c** number of its branch points in a 1-mm^2^ area. Whiskers represent the 10th and 90th percentiles, the box represents the 25th to 75th percentiles, the central line depicts the median, and the square inside each box indicates the average value. Each value was obtained from 30 images, which were captured from six independently repeated experiments (*n* = 30 images). **d** Observation of diffusion of 10-kDa FITC-dextran across the boundary face of a tube-like structure. Representative DIC and fluorescent images 2 h after addition of FITC-dextran. Scale bars, 100 µm. **e** The line profiles of the normalized fluorescence intensity in 62 locations (control) or 60 locations (pressure condition) across the boundary face of the tube-like structures from six experiments. **f** Ratio of fluorescence intensity between the inside and the outside of the tube-like structures shown as box-and-whisker plots, as defined in Fig. 1a (*n* = 62 [control] or 60 [pressure condition] locations). **g**, **h** Tubular robustness of cell-cell junctions formed under the pressure condition. **g** Representative fluorescence images of ZO-1 and VE-cadherin in the tube-liked structures. Scale bars, 100 µm. **h** Relative expression levels of ZO-1 and VE-cadherin in the tube-like structures (*right*) (mean + SEM, *n* = 7 experiments). The broad band apparent at approximately 65 kDa indicates bovine serum albumin derived from FBS contained in the experimental medium. **i** Proportions of ECs in the S and G2/M phases under the pressure condition (mean + SEM, *n* = 3 experiments). **j** Growth curve of ECs cultured sparsely after a 3-h exposure to hydrostatic pressure (mean ± SEM, *n* = 3 experiments). *p < 0.1, ***p < 0.01, NS: no significant difference (Welch’s *t* test; **b**, **c**, **f**, **h**).

We first examined the effect of hydrostatic pressure, mimicking the average increase in blood pressure (+50 mmHg) during exercise^11,12^, on the tubulogenic response of ECs. Human umbilical vein endothelial cells (HUVECs) formed tube-like structures after a 3-h pressure exposure and 13-h incubation, as observed under phase-contrast microscopy (Fig. 1a). In comparison with the control condition (i.e., 0 mmHg pressure), exposure to the hydrostatic pressure (+50 mmHg) promoted the formation of tube-like structures by HUVECs, with structures exhibiting significantly longer total length and more branch points in a 1-mm^2^ area (Fig. 1b, c). To evaluate the maturation of tube-like structures formed by HUVECs, we added 10 µg/mL of FITC-dextran into the collagen gel in which the tube-like structures were formed. FITC-dextran penetrated into the lumen of the tube-like structure after a 2-h incubation, as observed under differential interference contrast (DIC) and confocal laser-scanning microscopy (Fig. 1d). The relative fluorescence intensity of FITC-dextran dropped across the boundary face of the tube-like structures, approaching approximately half of the outside intensity at the center of the tubes under both control and pressure conditions (Fig. 1e). The ratio of the average fluorescence intensities outside (*I*_out_) and inside (*I*_in_) the tube-like structures was not affected by exposure to pressure (Fig. 1f). Hydrostatic pressure exposure did not, therefore, affect the maturation of the tube-like structures formed by HUVECs. To further examine the effects of hydrostatic pressure on tube-like structure formation, we analyzed the expression of cell-cell junction proteins in the tube-like structures. Tight junctions (ZO-1) and adherens junctions (VE-cadherin) formed in the tube-like structures after a 3-h pressure exposure and 13-h incubation, as observed under confocal laser-scanning microscopy (Fig. 1g). Pressure exposure did not affect the expression of VE-cadherin, whereas the expression of ZO-1 increased with marginal significance under the pressure condition (Fig. 1h and Supplementary Fig. 17). Hydrostatic pressure, therefore, marginally increases the robustness of the tube-like structures formed by HUVECs.

In highly confluent HUVECs, hydrostatic pressure advanced the cell cycle (Fig. 1i). Such premature cell cycle progression under positive pressure has been observed in other studies^14,17^ as well. The percentages of cells in the S or G2/M phases in the static culture and control conditions were similar (20% or less of total cells), with most cells remaining in the G1 phase. In contrast, after cells were exposed to pressure, the percentage of cells in the S phase peaked at 3 h and then decreased. The percentage of cells in the G2/M phases also reached a maximum (about 35%) at 6 h. The premature progression of the cell cycle is hypothesized to begin just after exposure to pressure because of the duration of the S phase^18^. This hypothesis is supported by our finding from HUVECs demonstrating significant nuclear translocation of cyclin D1 (which regulates the G1 restriction point^19^) following a 1-h pressure exposure (Supplementary Fig. 1). However, the effect of hydrostatic pressure on cell cycle progression lasted only 3 to 6 h after pressure exposure because the HUVECs adapted to the applied hydrostatic pressure stimulation of between 3 to 6 h^20^. Even if HUVECs were cultured under sparsely distributed conditions, their proliferation was enhanced by hydrostatic pressure. The application of pressure resulted in a 160% increase in the number of cells in the first 24 h of incubation, followed by relatively slow growth rates of 36% and 22% in the second and third 24 h of incubation, respectively (Fig. 1j). These data thus demonstrate that exposure to hydrostatic pressure transiently promotes endothelial tubulogenic responses of ECs, including proliferation.

### Pressure-induced Ras-ERK signaling leads to tube formation

**Fig. 2:**
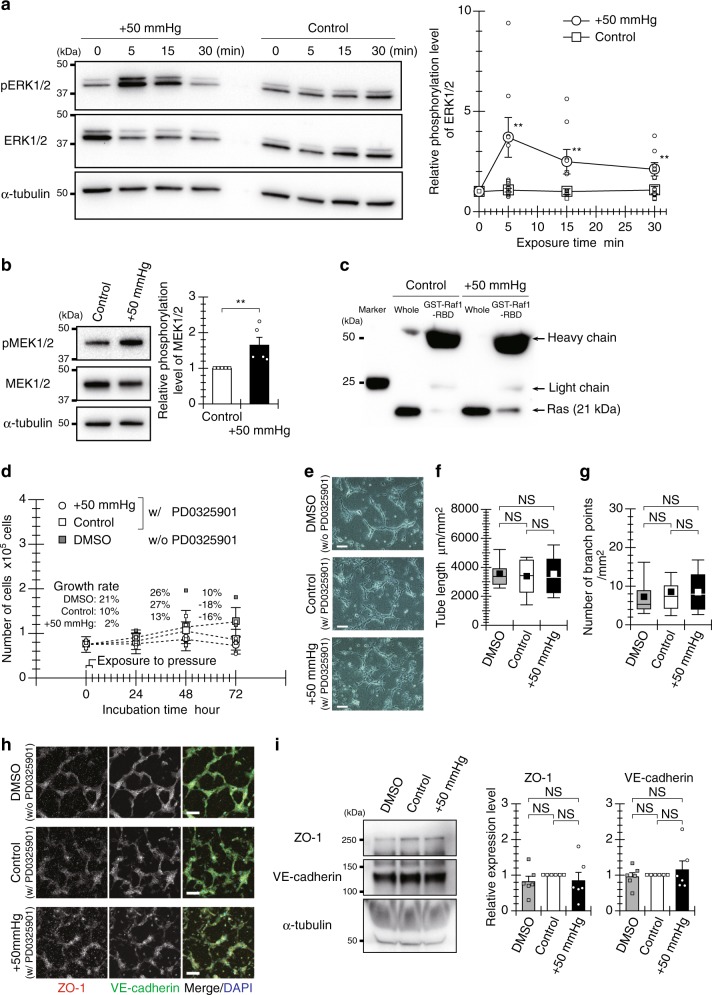
The Ras/ERK pathway is essential for hydrostatic pressure-induced endothelial tube formation. **a** ERK1/2 activation in HUVECs exposed to hydrostatic pressure, expressed as the relative intensity of p-ERK1/2 to that of ERK1/2 (mean ± SEM, *n* = 8 experiments). **b** MEK1/2 activation in ECs after a 5-min pressure exposure, expressed as the relative intensity of p-MEK1/2 to that of MEK1/2 (mean + SEM, *n* = 5 experiments). **c** Ras activity (RBD pull-down) in ECs after a 5-min pressure exposure (*n* = 3 experiments). **d** Growth curve of ECs cultured sparsely after a 3-h exposure to hydrostatic pressure in the presence of an MEK inhibitor (PD0325901) (mean ± SEM, *n* = 3 experiments). **e** Endothelial tube formation under the pressure condition in the presence of an MEK inhibitor (PD0325901). Scale bars, 100 µm. **f**, **g** Quantified total length and number of tube-like structure branch points in a 1-mm^2^ area. Each value is shown as a box-and-whisker plot, obtained from 25 images in five independently repeated experiments (*n* = 25 images). **h**, **i** Tubular robustness of cell-cell junctions formed under the pressure condition in the presence of an MEK inhibitor (PD0325901). **h** Representative fluorescence images of ZO-1 and VE-cadherin in the tube-liked structures. Scale bars, 100 µm. **i** Relative expression levels of ZO-1 and VE-cadherin in the tube-like structures (mean + SEM, *n* = 6 experiments). The broad band apparent at approximately 65 kDa indicates bovine serum albumin derived from FBS contained in the experimental medium. **p < 0.05 (Welch’s *t* test; **a**, **b**). NS: no significant difference (Tukey-Kramer test; **f**, **g**, **i**).

We then investigated the signaling pathway through which hydrostatic pressure induces angiogenesis, focusing on activation of the Ras/ERK pathway, which is strongly correlated with the EC proliferation associated with angiogenesis^21^. Hydrostatic pressure caused transient activation of ERK1/2 in HUVECs, with phosphorylation peaking within 5 min and then gradually returning to baseline level after 30 min (Fig. 2a and Supplementary Fig. 17). After a 5-min pressure exposure, the cells exhibited higher mean fluorescence intensity, indicating an approximately 1.5- and 2-fold increase in ERK1/2 activation in the cytoplasm and nucleus, respectively (Supplementary Fig. 2). The cells also exhibited a higher nuclear/cytoplasm ratio of activated ERK1/2, as compared with control. Hydrostatic pressure also induced phosphorylation of mitogen-activated protein kinase 1/2 (MEK1/2) (Fig. 2b and Supplementary Fig. 17) and clearly increased association of activated Ras and Raf-1 (Fig. 2c and Supplementary Fig. 17), as preliminary steps to ERK activation. Ras protein, a small guanosine triphosphatase (GTPase), functions as a master regulator of cell signaling^22^. Ras induces activation of MEK and its downstream ERK via interaction with the Ras effector Raf-1 (Ras/ERK pathway;^22,23^). Hydrostatic pressure, therefore, induces activation of the Ras/ERK pathway.

To further examine the relationship between activation of the Ras/ERK pathway and pressure-promoted tubulogenesis, we evaluated the EC proliferation and the formation of tube-like structures when ERK activation was inhibited using an MEK inhibitor. The pressure-enhanced proliferation was not observed under the inhibition of ERK activation (Fig. 2d). Although HUVECs formed tube-like structures in the presence of the inhibitor, a large proportion of the formed tube network exhibited short segments in both control and pressure-exposed cells (Fig. 2e). In addition, no significant differences were observed in the total length or number of branch points of the tube-like structures in a 1-mm^2^ area (Fig. 2f, g). Inhibition of ERK activation did not affect the maturation of the tube-like structures (Supplementary Fig. 3). The expression of ZO-1, which was marginally enhanced by pressure exposure, was not observed in cells treated with the MEK inhibitor (Fig. 2h, i, and Supplementary Fig. 17). These results suggest that hydrostatic pressure promotes endothelial tubulogenesis via the Ras/ERK pathway.

### Pressure-activated PKC via GPCRs drives Ras-ERK signaling

**Fig. 3:**
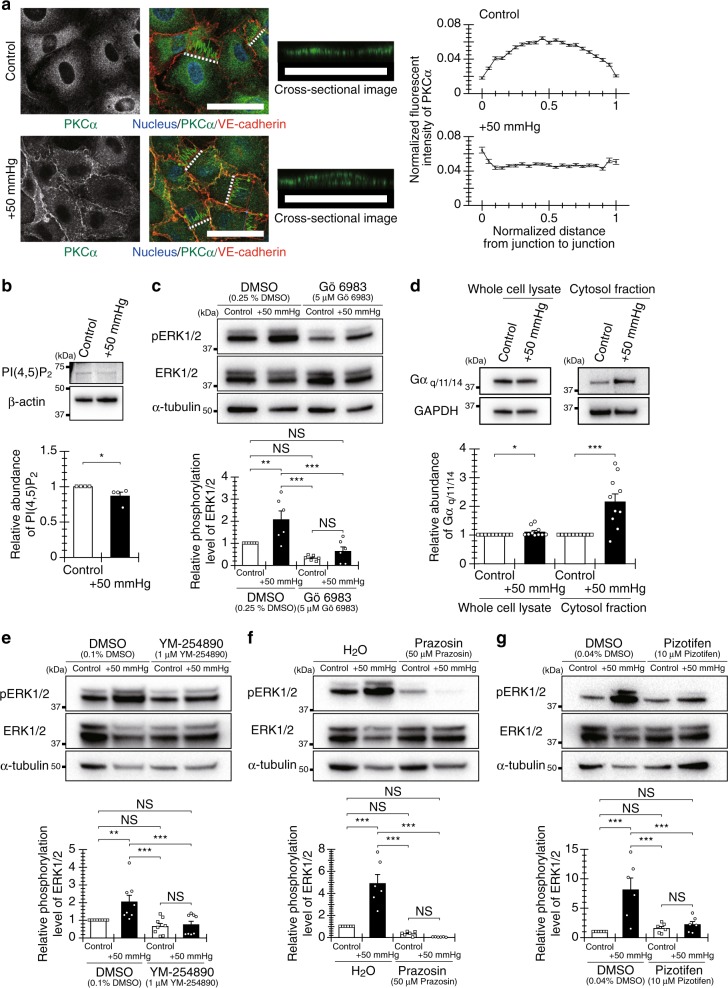
Activation of PKC via specific GPCRs drives hydrostatic pressure–induced activation of the Ras/ERK pathway in HUVECs. **a** Membrane translocation of activated PKC after a 5-min exposure to hydrostatic pressure, with quantified localization in 100 cells in four independently repeated experiments (*n* = 100 cells). Scale bars, 50 µm. **b** PI(4,5)P_2_ expression level after a 5-min pressure exposure (*n* = 4 experiments). **c** ERK1/2 activation after a 5-min pressure exposure in the presence of a PKC inhibitor (Gö6983) (*n* = 6 experiments). **d** Release of the G_q_ alpha subunit from the membrane to the cytoplasm after a 5-min pressure exposure (*n* = 10 experiments). ERK1/2 activation after a 5-min pressure exposure in the presence of **e** a G_q_ inhibitor (YM-254890) (*n* = 8 experiments), **f** an α1-AR antagonist (prazosin) (*n* = 6 experiments), or **g** an SR-2A antagonist (pizotifen) (*n* = 6 experiments). All data are presented as the mean ± SEM. *p < 0.1, **p < 0.05 (Welch’s *t*-test; **b**, **d**). **p < 0.05, ***p < 0.01, NS: no significant difference (Tukey-Kramer test; **c**, **e**–**g**).

We then sought to determine what drives the hydrostatic pressure–induced activation of the Ras/ERK pathway. Although the vascular endothelial growth factor receptor 2 (VEGFR2)/phospholipase C (PLC) pathway is known to regulate Ras/ERK signaling^24^, hydrostatic pressure did not induce tyrosine phosphorylation of VEGFR2 in our study (Supplementary Fig. 4 and Supplementary Fig. 17). However, PKC, an activator of the Ras/ERK pathway^25^, was activated in HUVECs exposed to hydrostatic pressure, as observed by its relocation from the cytoplasm to the cell membrane (Fig. 3a). Three major isoforms of PKC have been identified (i.e., conventional, novel, and atypical), with activation requiring calcium ion (Ca^2+^) or diacylglycerol (DAG), depending on the isoform^26^. Exposure of HUVECs to hydrostatic pressure did not induce noticeable differences in the intracellular Ca^2+^ concentration relative to control, although a slight decrease in membrane potential was observed (Supplementary Fig. 5). The concentration of phosphatidylinositol 4,5-bisphosphate (PI[4,5]P_2_), which is hydrolyzed to inositol trisphosphate and DAG by PLC^27^, tended to decrease after exposure to hydrostatic pressure (Fig. 3b and Supplementary Fig. 17), with concomitant activation of PKC and the Ras/ERK pathway. These experimental data were supported by the following observations: (i) in the presence of inhibitors of PLC (Supplementary Fig. 6 and Supplementary Fig. 17) or PKC (Fig. 3c and Supplementary Fig. 17), ERK was not activated even in pressure-exposed cells; and (ii) in the presence of a specific inhibitor of PKCα/β (Supplementary Fig. 7 and Supplementary Fig. 17), there was no difference in the level of ERK activation between control and pressure-exposed cells, although the level in pressure-exposed cells was still not significantly different in comparison with that in pressure-exposed cells not treated with the inhibitor.

We then confirmed the pressure-associated activation of G_q_ protein (i.e., release of the G_q_ alpha subunit from the cell membrane to the cytoplasm), which is known to activate PLC^28^ (Fig. 3d and Supplementary Fig. 17). Inhibition of G_q_ protein activation prevented pressure-induced ERK activation (Fig. 3e and Supplementary Fig. 17). The activation of G_q_ protein is regulated by GPCRs. We investigated the relationship between pressure-induced ERK activation and four GPCRs to which G_q_ protein binds (i.e., α1-adrenergic receptor [α1-AR], angiotensin II type I receptor [AT1-R], histamine H1 receptor [H1-R], and serotonin receptor type 2A [SR-2A]) and that are known to be expressed in HUVECs (Supplementary Fig. 8 and Supplementary Fig. 17). Inhibition of GPCRs using antagonists for α1-AR and SR-2A prevented pressure-induced ERK activation (Fig. 3f, g, Supplementary Fig. 9, and Supplementary Fig. 17), suggesting that activation of PKC via α1-AR and SR-2A drives the hydrostatic pressure–induced activation of the Ras/ERK pathway. This notion is supported by the findings that inhibition of G protein activation prevented pressure-induced translocation of PKC (Supplementary Fig. 10).

To further examine the relationship between activation of PKC via specific GPCRs and pressure-promoted tubulogenesis, we evaluated the formation of tube-like structures when the activations of PKC, G protein, and GPCR were inhibited using each inhibitor. Inhibition of their activation prevent pressure-induced increases in the length of the tube-like structures and the number of their branch points (Supplementary Fig. 11). These results suggest that hydrostatic pressure promotes endothelial tubulogenesis via the Ras/ERK pathway driven by the activation of PKC, G protein, and specific GPCRs.

### Aquaporin-mediated water efflux activates Ras-ERK signaling

**Fig. 4:**
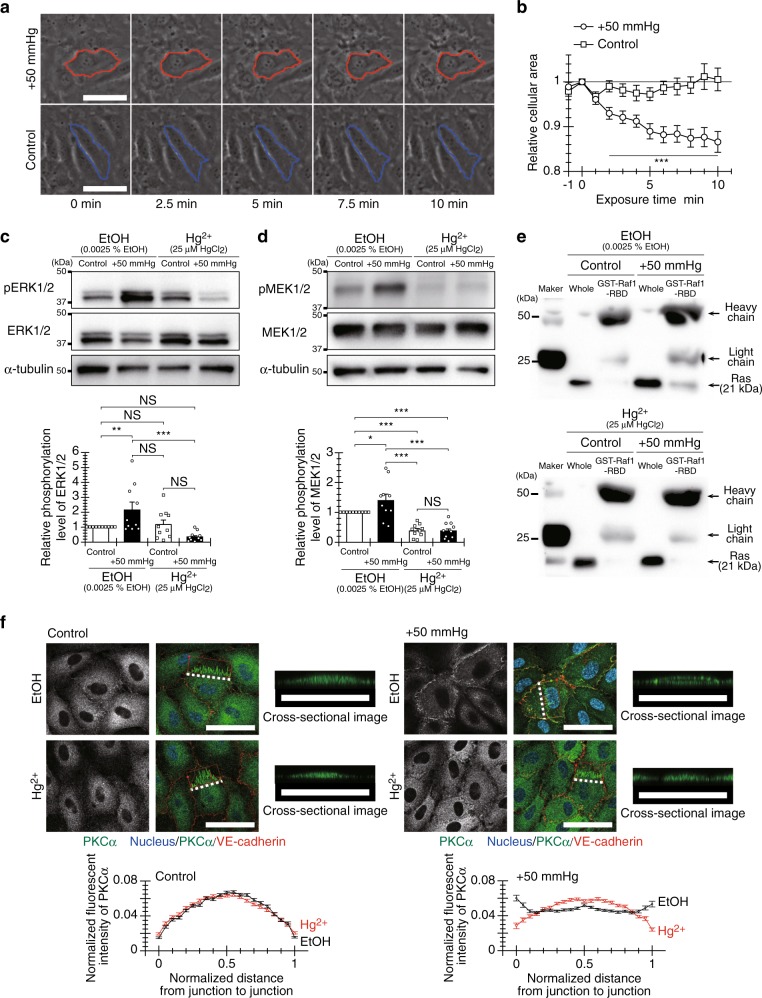
AQP1-mediated water efflux plays a key role in hydrostatic pressure–induced activation of the Ras/ERK pathway in HUVECs. **a** Time sequence phase-contrast images depicting cell contraction and **b** changes in relative cell area under the pressure condition. Each value was obtained from 30 cells, which were captured in five independently repeated experiments (*n* = 30 cells). Scale bars, 50 µm. **c** ERK1/2 activation (*n* = 10 experiments), **d** MEK1/2 activation (*n* = 10 experiments), and **e** Ras activity (*n* = 3 experiments) after a 5-min exposure to hydrostatic pressure with inhibition of AQP1-mediated water influx and efflux using HgCl_2_. **f** Membrane translocation of activated PKC in HUVECs after a 5-min pressure exposure and quantified localization in 100 cells in four independently repeated experiments (*n* = 100 cells) with inhibition of AQP1-mediated water flux using HgCl_2_. Scale bars, 50 µm. All data are presented as the mean ± SEM. ***p < 0.01 (Welch’s *t* test; b). *p < 0.1, **p < 0.05, ***p < 0.01, NS: no significant difference (Tukey-Kramer test; **c**, **d**).

Finally, we investigated how HUVECs sense hydrostatic pressure and convert it to a biochemical signal that leads to the activation of PKC via GPCRs. We hypothesized that pressure causes an efflux of water from cells, based on a kinetic model of water^29^ in which flux is defined by the difference between hydrostatic and osmotic pressures across the cell membrane. This hypothesis is supported by our findings indicating cell contraction (Fig. 4a, b, and Supplementary Movie 1 and 2) and the efflux of a fluorescent Ca^2+^ indicator (Supplementary Fig. 12a and 12b) under the pressure condition. Similar cell contraction is reportedly caused by hydrostatic pressure^30^. AQP1 is a water channel molecule that enhances membrane water permeability^31^. Although translocation of AQP1 to the cell membrane is reportedly induced by osmotic stimulation^32^, our results did not demonstrate this (Supplementary Fig. 13 and Supplementary Fig. 17). We therefore examined the inhibition of water flux through AQP1. Following inhibition of AQP1 using mercuric (II) chloride (HgCl_2_)^33^, no activation of the Ras/ERK pathway was observed, even in cells exposed to pressure (Fig. 4c–e, and Supplementary Fig. 17). In addition, no pressure-induced PKC activation was observed in cells in which water flux was inhibited (Fig. 4f). Cells, in which water flux was inhibited, exhibited no contraction (Supplementary Fig. 14 and Supplementary Movies 3, 4, 5, and 6) and no efflux of the fluorescent Ca^2+^ indicator, and simultaneously, pressure exposure did not induce an increase in the intracellular Ca^2+^ ion concentration (Supplementary Fig. 12c, 12d, 12e, and 12f). Based on these results, we conclude that AQP1-mediated water efflux plays a key role in the hydrostatic pressure–induced activation of PKC via α1-AR and SR-2A and activation of the Ras/ERK pathway that ultimately leads to tubulogenesis. These findings support the hypothesis that water efflux via AQP1 converts hydrostatic pressure to biochemical signals that ultimately activate PKC through GPCRs.

## Discussion

In this study, we elucidated a part of the mechanism by which hydrostatic pressure promotes endothelial tube formation. This finding provides a potential to promote endothelial tubulogenesis by controlling hydrostatic pressure in vivo. Our results answer in part the long-standing question as to how ECs sense hydrostatic pressure and convert it to intracellular biochemical signals (Supplementary Fig. 15). Although we could not determine the mechanism by which AQP1-mediated water efflux activates GPCRs, we believe that contraction of the cell membrane resulting from the efflux of water is important in GPCR activation. We expect that in addition to promoting tubulogenesis, hydrostatic pressure also plays a crucial role in the pathology of a variety of diseases (mechanopathology). By better understanding the effects of hydrostatic pressure, we could ultimately develop methods to manipulate it and thus improve human health (mechanotherapy).

Pressure-enhanced endothelial proliferation leading to tubulogenic responses was confirmed in our previous studies^17,30^. Hydrostatic pressure induces the forcible progression of the stagnant cell cycle in ECs via contact inhibition without morphologic changes such as elongation or altered orientation^17^. We also demonstrated the importance of actomyosin contractility on cell contraction induced by hydrostatic pressure^30^. However, our previous studies did not clarify the detailed mechanisms linking these cellular responses to endothelial tubulogenesis (i.e., pressure-induced signal transduction leading to tubulogenesis). Sustained pressure reportedly promotes sprout angiogenesis from spheroids composed of bovine aorta ECs^16^. Pressure-sensitive upregulation of VEGF-C and VEGFR3 expression plays a critical role in this sprout angiogenesis in the presence of growth factors such as fibroblast growth factor (FGF) or VEGF. Notably, in the present study, hydrostatic pressure promoted tubulogenic responses even in the absence of FGF and VEGF. Pressure-promoted endothelial tube formation and pressure-induced signal transduction, which were demonstrated in the present study, differ from angiogenesis induced via the commonly known VEGFR pathway^24,34^.

The elucidated mechanism by which hydrostatic pressure promotes endothelial tube formation is based on tube formation reproduced by cultured HUVECs in vitro. Given that tumor angiogenesis is regulated by tumor interstitial fluid pressure^35,36^ and sprouting angiogenesis is controlled by vascular internal pressure^37^ in vivo, endothelial tubulogenesis can be promoted by pressure in vivo via the elucidated mechanism. However, some details of the mechanism of pressure-promoted tubulogenesis remain unclear, as we adopted artificial conditions in the present study, such as the use of fetal bovine serum (FBS)-free medium and only one pressure condition. Additional investigation regarding potential side effects of the inhibitors is also needed, as these inhibitors interact with a variety of cellular molecules, even though we examined their concentration and incubation time with regard to cytotoxicity and overreaction with target molecules. A few inhibitors suppressed both ERK1/2 phosphorylation and activity. Further in vitro and in vivo studies are therefore needed in order to address these issues and fully elucidate the mechanism by which hydrostatic pressure promotes endothelial tubulogenesis.

## Methods

### Chemicals and antibodies

All chemicals used as inhibitors and antagonists for target proteins are indicated in Supplementary Table 1. Primary and secondary antibodies used in this study are described in Supplementary Tables 2 and 3.

### Cell culture

HUVECs (lot nos. 2818 [black donor] and 2840 [Caucasian donor], 200–05n, Cell Applications, San Diego, CA, USA) were cultured in Medium 199 (M199; 31100–035, Gibco, Thermo Fisher Scientific, Waltham, MA, USA) containing 20% heat-inactivated FBS (12483–020, Gibco or 04–001–1 A, Biological Industries, Beit-Haemek, Israel), 10 µg/L human basic fibroblast growth factor (bFGF; GF-030–3, Austral Biologicals, San Ramon, CA, USA), and 1% penicillin/streptomycin (P/S; 15140–122, Gibco). HUVECs from the fourth to ninth passages were used for experiments in this study. The experiments were conducted using three types of experimental medium (EM): M199 containing 10% heat-inactivated FBS and 1% P/S (EM1), FBS-free M199 (EM2), and a FBS-free M199 with Hank’s salts (M0393, Sigma-Aldrich, St. Louis, MO, USA) (EM3).

### Exposure to hydrostatic pressure

HUVECs cultured in dishes were exposed to hydrostatic pressure using a system reported in our previous work^17^. The system device was filled with EM, and pressure was the applied to ECs by compressing the volume of the EM. The system was maintained at 37 °C in a CO_2_-supplied incubator. Cells were exposed to a hydrostatic pressure of 0 (control) or +50 mmHg (pressure condition). The pressure value was set up in accordance with the average increase in blood pressure (+50 mmHg) during exercise^11,12^.

For imaging living cells, HUVECs were exposed to hydrostatic pressure (+50 mmHg) using a custom-made hydrostatic pressure microscopy system (Supplementary Fig. 16) consisting of a cell culture dish, polycarbonate pressure chamber, silicone gasket, O-ring, quartz glass, two ball valves, a thermostatic chamber, syringe pump, and wide-field fluorescence microscope (EVOS FL Cell Imaging System, Thermo Fisher Scientific) or confocal laser-scanning microscope (LSM800, Carl Zeiss, Oberkochen, Germany). This system allows for observations using both epi-fluorescence and transmitted light.

### Tube formation assay

Tube formation assays were performed with reference to the study by Deroanne et al.^38^, with slight modifications. Collagen gels (300 µL each) were formed on 35-mm diameter glass-based dishes (3910–035, AGC Techno Glass, Shizuoka, Japan) by mixing ice-cold collagen solution (4.0 mg/mL; 10× M199, H_2_O, native collagen [IAC-50, KOKEN, Tokyo, Japan], 10 mM NaHCO_3_, 10 mM HEPES-NaOH, pH 7.5) and incubating for 30 min at 37 °C. HUVECs were seeded on the gels at a density of 1.2×10^5^ cells/cm^2^ and incubated in EM1 for 2 h to facilitate spreading. When cells reached 100% confluency, the EM1 was then removed and the HUVECs were covered with overlaying collagen gel (200 µL). After gelation for 15 min at 37 °C, the collagen gel layers were placed inside the pressure exposure system, and the cells between the layers were exposed to pressure in EM1 for 3 h. The cells were then removed from the system and incubated in a CO_2_ incubator for 13 h. After incubation, the cells were fixed in 4% paraformaldehyde phosphate buffer solution (PFA; 163–20145, Wako Pure Chemical Industries, Osaka, Japan) for 30 min at room temperature. For the inhibition study, inhibitor was added to EM1 and the cells incubated for 30 min before overlaying of the collagen gel. Tube-like structures formed by HUVECs were observed using an inverted phase-contrast microscope (Ti-U, Nikon, Tokyo, Japan) or a wide-field fluorescence microscope (EVOS FL Auto 2 Imaging System, Thermo Fisher Scientific).

### Tube maturation and robustness assays

Maturation of the tube-like structures formed by HUVECs was monitored using FITC-dextran (10 kDa, F0918, Tokyo Chemical Industry, Tokyo, Japan). Tube-like structures in collagen gel were first incubated in EM1 containing 10 µg/mL FITC-dextran for 2 h, which was sufficient time to allow diffusion into the gel and reaching of steady state^39^. After incubation, images of horizontal sections of the tube-like structures were captured using DIC and confocal laser-scanning microscopy (LSM800, Carl Zeiss). Focusing on cell-cell junction proteins, the robustness of the tube-like structures was evaluated using immunofluorescence staining and immunoblotting. For immunofluorescence staining, the formed tube-like structures were fixed with PFA for 30 min, followed by staining using primary and secondary antibodies. A whole-cell lysate was obtained by collecting the supernatant after washing with ice-cold phosphate-buffered saline (PBS; 05913, Nissui Pharmaceutical, Tokyo, Japan), picking up the whole set of collagen gels including the tube-like structures using 4× Laemmli sample buffer (161–0747, Bio-Rad Laboratories, Hercules, CA, USA), homogenizing by vigorous shaking, and centrifugation at 21,500*g* for 15 min. Dithiothreitol (DTT; 161–0611, Bio-Rad Laboratories) was added to the collected whole-cell lysates to a final concentration of 20 mM, and the lysates were then boiled for 5 min. The whole-cell lysates were analyzed by SDS-PAGE followed by immunoblotting to detect cell-cell junction proteins (i.e., ZO-1 and VE-cadherin).

### Cell cycle analysis

HUVECs were cultured in 60-mm diameter plastic dishes (MS-11600, Sumitomo Bakelite, Tokyo, Japan) pre-coated with 0.1% bovine gelatin solution (G9391, Sigma-Aldrich). After reaching high confluence (100%), the HUVECs were washed twice and incubated with EM1 for 3 h to wash out bFGF. The cells were then exposed to hydrostatic pressure for 3, 6, 12, or 24 h, harvested from the dish using 0.05% trypsin-EDTA (25300–054, Gibco), and centrifuged for 5 min at 185*g* after inactivation of the trypsin-EDTA using EM1. The collected cells were then washed with PBS and fixed in 70% ice-cold ethanol. After another PBS wash, the cell density was adjusted to 500 cells/µL. Nuclear DNA was stained using Guava Cell Cycle reagent (4500–0220, Merck Millipore, Darmstadt, Germany) for 30 min. The fluorescence intensity of 5000 cells was measured, and the percentage of HUVECs in each phase of the cell cycle was determined using flow cytometry (Guava easyCyte 6HT, Merck Millipore).

### Cell proliferation assay

A total of 8 × 10^4^ HUVECs were seeded in a 60-mm diameter plastic dish coated with 0.1% gelatin. After incubation for 1 h, the cells were exposed to pressure in EM1 for 3 h, then incubated in a CO_2_ incubator for 24, 48, or 72 h, after which the cells were harvested from the dish using 0.05% trypsin-EDTA and centrifuged for 5 min at 1000 rpm after inactivation of the trypsin-EDTA with EM1. The cells were resuspended in EM1 (200 µL) and stained with Guava ViaCount reagent (4000–040, Merck Millipore) for 10 min or trypan blue solution (15250–061, Gibco). The number of live cells was then determined using flow cytometry or a hemocytometer (Burker-Turk).

### Protein activation assay

HUVECs were cultured in a 35-mm diameter glass-bottom dish, a 35-mm diameter plastic dish (3000–035, AGC Techno Glass), or a 60-mm diameter plastic dish, each pre-coated with 0.1% bovine gelatin. Highly confluent HUVECs were washed twice with FBS-free EM2 and incubated in the same medium for 3 h to wash out bFGF and starve the cells. Cells were then exposed to pressure for 5, 15, and 30 min or 1 h, collected as described above, and then examined by immunoblotting or immunofluorescence staining. Inhibitors and antagonists were introduced into the EM2 after 3 h of FBS starvation, and the cells were then incubated for the times indicated in Supplementary Table 1 before exposure to pressure.

### Immunofluorescence staining

After exposure to hydrostatic pressure, HUVECs were fixed with 4% PFA at room temperature or ice-cold methanol at −20 °C in accordance with the data sheets for the antibodies used. The cells were permeabilized with 0.1 or 0.3% TritonX-100 in PBS and incubated in 1% Block Ace (BA; UKB40, DS Pharma Biomedical, Osaka, Japan) in PBS to prevent nonspecific antibody adsorption. The cells were then stained using the primary and secondary antibodies diluted in 1% BA in PBS and PBS, respectively, at predefined concentrations (Supplementary Tables 2 and 3). Cell nuclei were stained using 4ʹ,6-diamidino-2-phenylindole (DAPI; D1306, Thermo Fisher Scientific). Stained HUVECs were observed using a wide-field fluorescence microscope (Axio Observer D1, Carl Zeiss) or an inverted confocal laser-scanning microscope (LSM800, Carl Zeiss).

### Cellular fractionation

Cytosolic and crude cell membrane fractions were prepared according to the following protocol. Cells were washed twice with ice-cold PBS, scraped from the surface, transferred to microtubes with ice-cold hypotonic buffer (7.5 mM Na_2_HPO_4_, 1 mM EDTA, protease inhibitor cocktail [P8340, Sigma-Aldrich]), and homogenized by passage through a 25 G needle (NN-2516R, Terumo, Tokyo, Japan). The cytosolic fraction was obtained by collecting the supernatant after two consecutive centrifugations (500*g* at 4 °C for 5 min followed by 20,000*g* at 4 °C for 30 min). Proteins were recovered from the cytosolic fraction in 2× Laemmli sample buffer (161–0737, Bio-Rad Laboratories). The pellet after the second centrifugation was resuspended in modified Laemmli buffer (65 mM Tris-HCl [pH 7.5], 0.1 mM EGTA, 0.1 mM EDTA, 1 mM Na_3_VO_4_, 1 mM NaH_2_PO_4_, 10% glycerol, 2% SDS, 20 mM DTT, and protease inhibitor cocktail), incubated on ice for 5 min, and homogenized by vigorous shaking. The crude cell membrane fraction was obtained by collecting the supernatant after centrifugation at 21,500*g* for 10 min. The whole-cell lysate was obtained by collecting the supernatant after the ice-cold PBS washing, scraping the cells using the modified Laemmli buffer, and centrifugation at 21,500*g* for 10 min.

### Pull-down assay

HUVECs were washed with ice-cold Tris-buffered saline (TBS; 25 mM Tris-HCl [pH 7.5], 150 mM NaCl), lysed using lysis buffer (25 mM Tris-HCl [pH 7.2], 150 mM NaCl, 5 mM MgCl_2_, 1% NP-40, 5% glycerol, and protease inhibitor cocktail), scraped, and collected in a microtube. After a 5-min incubation on ice, cell debris was removed by centrifugation at 16,000*g* at 4 °C for 15 min. The pull-down assay was conducted using an Active Ras Pull-Down and Detection kit (16117, Thermo Fisher Scientific) according to the manufacturer’s instructions, and proteins were recovered from the resultant immunoprecipitates in 2× SDS sample buffer.

### Immunoblotting

Samples were subjected to SDS-PAGE and then transferred onto an Immun-Blot PVDF membrane (162–0177, Bio-Rad Laboratories). The membrane was blocked with TBS containing 1% BA and 0.05% Tween 20 and then stained using primary and secondary antibodies diluted in TBS containing 1% BA and 0.05% Tween 20 at predefined concentrations (Supplementary Tables 2 and 3). Can Get Signal Immunoreaction Enhancer Solution (NKB-101, Toyobo, Osaka, Japan) was added to the antibody diluent buffer as necessary. The blotted proteins were detected and visualized using Clarity Western ECL Substrate (170–5061, Bio-Rad Laboratories) or an AP Conjugate Substrate kit (170–6432, Bio-Rad Laboratories). Protein loading was monitored using loading control proteins (i.e., α-tubulin, β-actin, and GAPDH). The molecular weight of each protein was determined based on Precision Plus Protein Dual Color Standards (161–0374, Bio-Rad Laboratories). Membranes were stripped of bound antibodies and re-probed with different primary and secondary antibodies. Stripping was accomplished by soaking the membrane in stripping buffer (100 mM β-mercaptoethanol, 50 mM Tris-HCl [pH 6.8], and 2% SDS) at 50 °C for 30 min.

### Imaging of living cells exposed to hydrostatic pressure

HUVECs were grown to high confluence (100%) on 35-mm diameter glass-bottom dishes (3910–035-IN, AGC Techno Glass) coated with 0.1% bovine gelatin in FBS-free EM3 for 3 h before live imaging. In the custom-made hydrostatic pressure microscopy system, the intracellular Ca^2+^ ion concentration and cellular membrane potential were visualized using Fluo-8, AM (21082, AAT Bioquest, Sunnyvale, CA, USA) and bis(1,3-dibutylbarbituric acid)trimethine oxonol, sodium salt (DiBAC4[3]; D545, Dojindo Molecular Technologies, Kumamoto, Japan), respectively, according to the manufacturers’ instructions.

### Quantification of length and branch-point number of tube-like structures

The total length of the tube-like structures was measured by tracing the tube-like structures with the freehand lines tool, and the number of tube-like structure branch points was determined by counting them in phase-contrast images using ImageJ software (US National Institutes of Health) (Figs. 1a–c and 2e–g, and Supplementary Fig. 12).

### Maturation of tube-like structures

The maturation of tube-like structures was analyzed based on diffusion of FITC-dextran from the outside to the inside of the tube-like structures. We first prepared a fluorescent image minus background noise using ZEN software (Carl Zeiss) and stacked this image onto the corresponding DIC image using ImageJ software (Fig. 1d and Supplementary Fig. 4a). Line profiles of fluorescence intensity of FITC-dextran were obtained at a location across the boundary face of the tube-like structures, which was randomly selected on the DIC image. The line width for extracting the line profile was set to 20 pixels. The line profile was extracted from the measurements on a line with perpendicular to the boundary face. The location of the line profile was normalized by its length, and was shown in the range of −0.25 (center of the tube-like structure) to 0.25. The relative fluorescence intensity of FITC-dextran was calculated based on its intensity in the collagen gel where no tube-like structures were present (Fig. 1e and Supplementary Fig. 4b). For evaluation of tube-like structure maturation, the ratio of the averaged fluorescence intensities outside (*I*_out_) and inside (*I*_in_) the tube-like structures was calculated (Fig. 1f and Supplementary Fig. 4c).

### Nuclear/cytoplasm ratios of cyclin D1 and activated ERK

The nuclear/cytoplasm ratio of cyclin D1 or activated ERK was also determined using ImageJ software. Fluorescence in the nucleus was extracted by referring to the captured images of DAPI staining. The averaged fluorescence intensity in the nucleus and cytoplasm in the whole image was measured, and the relative averaged fluorescence intensity was then calculated as the ratio between the intensity of each sample and the averaged value for the entire sample. The relative averaged intensity between the nucleus and cytoplasm was finally determined as the nuclear/cytoplasm ratio (Supplementary Figs. 2 and 3).

### PKCα localization

Line profiles of fluorescence intensity were obtained for quantitative representation of PKCα localization, based on our previous work^40^, with slight modifications. Captured fluorescence images of PKCα were processed using ZEN Imaging software. The fluorescence intensity was determined over a distance covering the membrane and the cytoplasm on the image of a layer with the maximum intensity of VE-cadherin, which was selected from 20 z-stack images with 0.6-µm intervals. Relative PKCα localization was evaluated with the total amount of one line profile of the fluorescence intensity set to a value of 1 (Figs. 3a, 4f, and Supplementary Fig. 11).

### Cell area, Ca^2+^ ion concentration, and membrane potential

The cell area, Ca^2+^ ion concentration, and cellular membrane potential were assessed using ImageJ software. Cell area was measured by tracing the outer periphery of the cell based on the phase-contrast images (Fig. 4a, b, and Supplementary Fig. 15). The intracellular Ca^2+^ ion concentration and cellular membrane potential were quantified based on the integrated fluorescence intensity, which was obtained from the product of the averaged intensity and the selected cellular area of Fluo-8 and DiBAC_4_(3) in the cell, respectively, extracted referring to the maximum intensity projection of confocal microscopic images (Supplementary Fig. 6).

### Quantification of protein expression and phosphorylation

The density of protein bands on immunoblots was determined using Image Lab (170–9691, Bio-Rad Laboratories). The relative expression and phosphorylation levels of each protein were calculated with the control condition set to a value of 1 on the same membrane.

### Statistics and reproducibility

All values are shown as mean ± standard error (SEM) unless stated otherwise. Each data was obtained from at least three independently repeated experiments (Supplementary Data 1). Statistical significance was calculated using the two-sided Welch’s *t*-test for comparisons of two groups or the Tukey-Kramer test for multiple comparisons, with statistical significance set at *p* ≤ 0.1 (marginally significant), *p* ≤ 0.05, and *p* ≤ 0.01 (significant difference). The effect size of each statistical test was analyzed using the Pearson’s correlation coefficient *r*^41^, which is defined as follows:

$$r = \sqrt {\frac{{t^2}}{{t^2 + df}}}$$

Here, *t* and *df* represent the statistics and the degrees of freedom, respectively, and they were obtained from the following equations^42,43^:

$$t = \frac{{\bar X_i - \bar X_j}}{{\sqrt {\frac{{s_i^2}}{{N_i}} + \frac{{s_j^2}}{{N_j}}} }}$$

$$df \approx \frac{{\left( {\frac{{s_i^2}}{{N_i}} + \frac{{s_j^2}}{{N_j}}} \right)^2}}{{\frac{{s_i^4}}{{N_i^2\left( {N_i - 1} \right)}} + \frac{{s_j^4}}{{N_j^2\left( {N_j - 1} \right)}}}}$$

where $\bar X$, *s*, and *N* are the mean value, standard deviation, and size of sample, respectively. The exact *p*-values and the effect size for all statistically tested data are described in Supplementary Data 2.

### Reporting Summary

Further information on research design is available in the Nature Research Reporting Summary linked to this article.

## Supplementary information

Supplementary Information

Description of Additional Supplementary Files

Supplementary Movie 1

Supplementary Movie 2

Supplementary Movie 3

Supplementary Movie 4

Supplementary Movie 5

Supplementary Movie 6

Supplementary Data 1

Supplementary Data 2

Reporting Summary

## Data Availability

The authors declare that all data supporting the findings of this study are available within this article and its supplementary information files or from the corresponding author upon reasonable request.
