# Supplementary material for: Hydrostatic pressure promotes endothelial tube formation through aquaporin 1 and Ras-ERK signaling
Source: Commun Biol. 2020 Apr 2;3:152. doi: 10.1038/s42003-020-0881-9 (PMC7118103; doi:10.1038/s42003-020-0881-9)
Supplement: Supplementary file 1 — Supplementary Information [file 42003_2020_881_MOESM1_ESM.pdf]

Supplementary Information

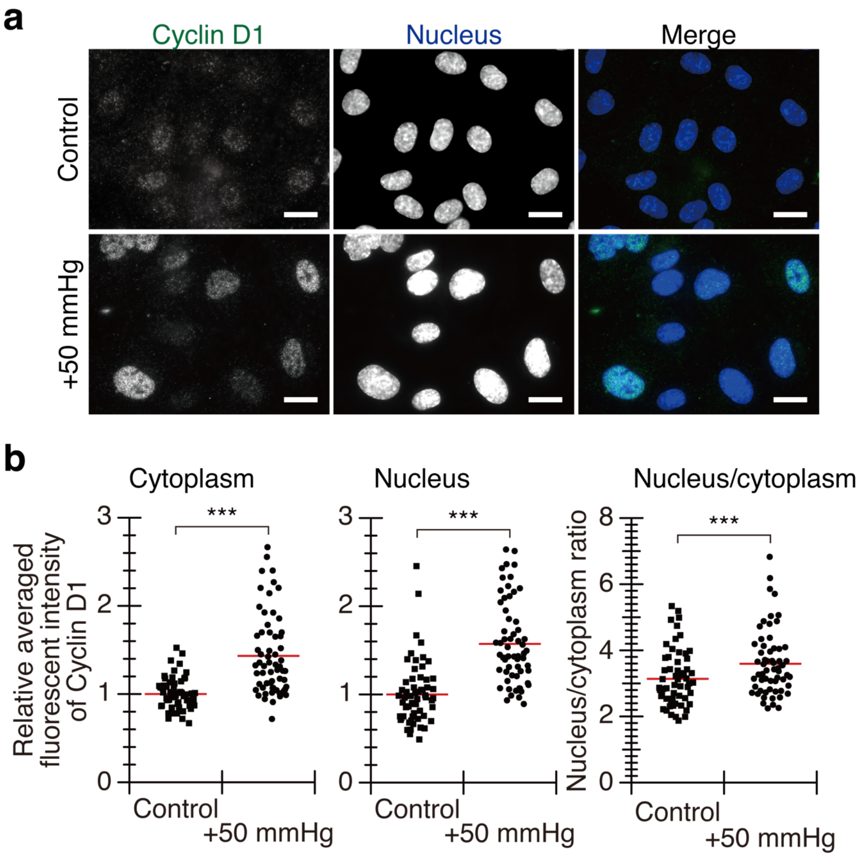

Supplementary Fig. 1.

Nuclear translocation of cyclin D1, a regulator of the G1 restriction point, induced by hydrostatic pressure. (**a**) Localization of cyclin D1 in HUVECs after a 1-h exposure to hydrostatic pressure. Scale bars, 20 μm. (**b**) Quantification of cyclin D1 localization in the cytoplasm or nucleus and nuclear/cytoplasm ratio. The quantified results indicate the averaged fluorescence intensities of cyclin D1 in the cytoplasm and nucleus, respectively. Each value was obtained from 60 images (control) or 61 images (pressure condition), which were captured in three independently repeated experiments (n = 60 [control] or 61 [pressure condition] images). Each bold bar represents the average value. ***$p < 0.01$ (Welch's t test).

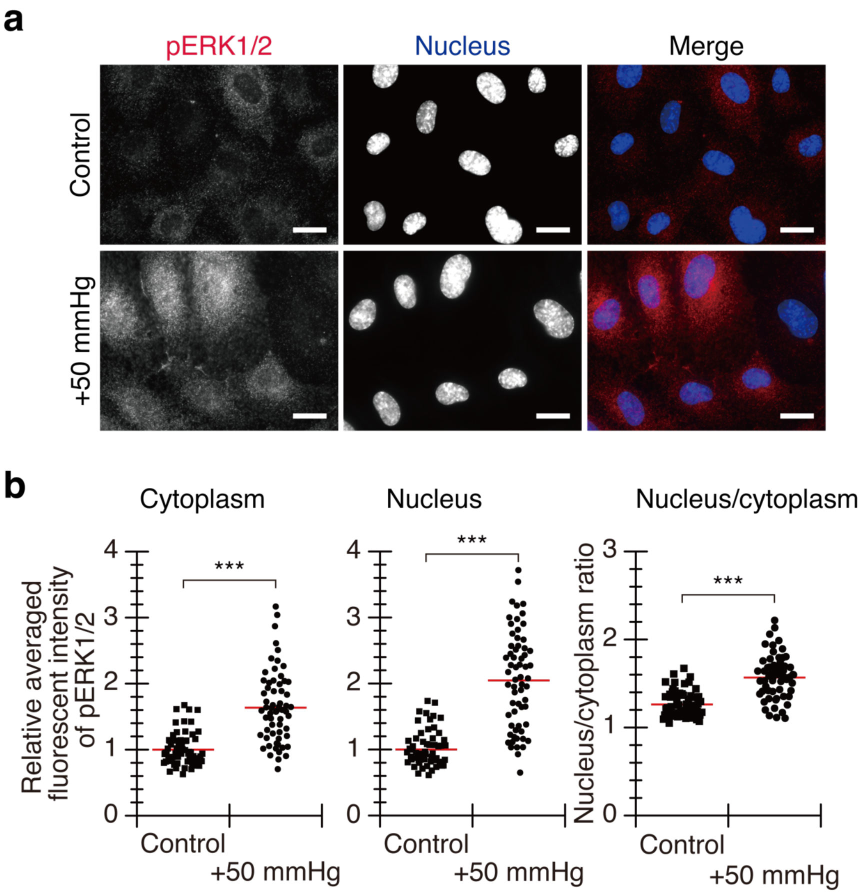

Supplementary Fig. 2.

Nuclear translocation of ERK1/2 activated by hydrostatic pressure. (**a**) Localization of activated ERK1/2 in ECs after a 5-min exposure to hydrostatic pressure. Scale bars, 20 μm. (**b**) Quantification of activated ERK1/2 localization in the cytoplasm or nucleus and nuclear/cytoplasm ratio. The quantified results indicate the averaged fluorescence intensities of activated ERK1/2 in the cytoplasm and nucleus, respectively. Each value was obtained from 62 images (control) or 63 images (pressure condition), which were captured in three independently repeated experiments (n = 62 [control] or 63 [pressure condition] images). Each bold bar represents the average value. ***$p < 0.01$ (Welch's t test).

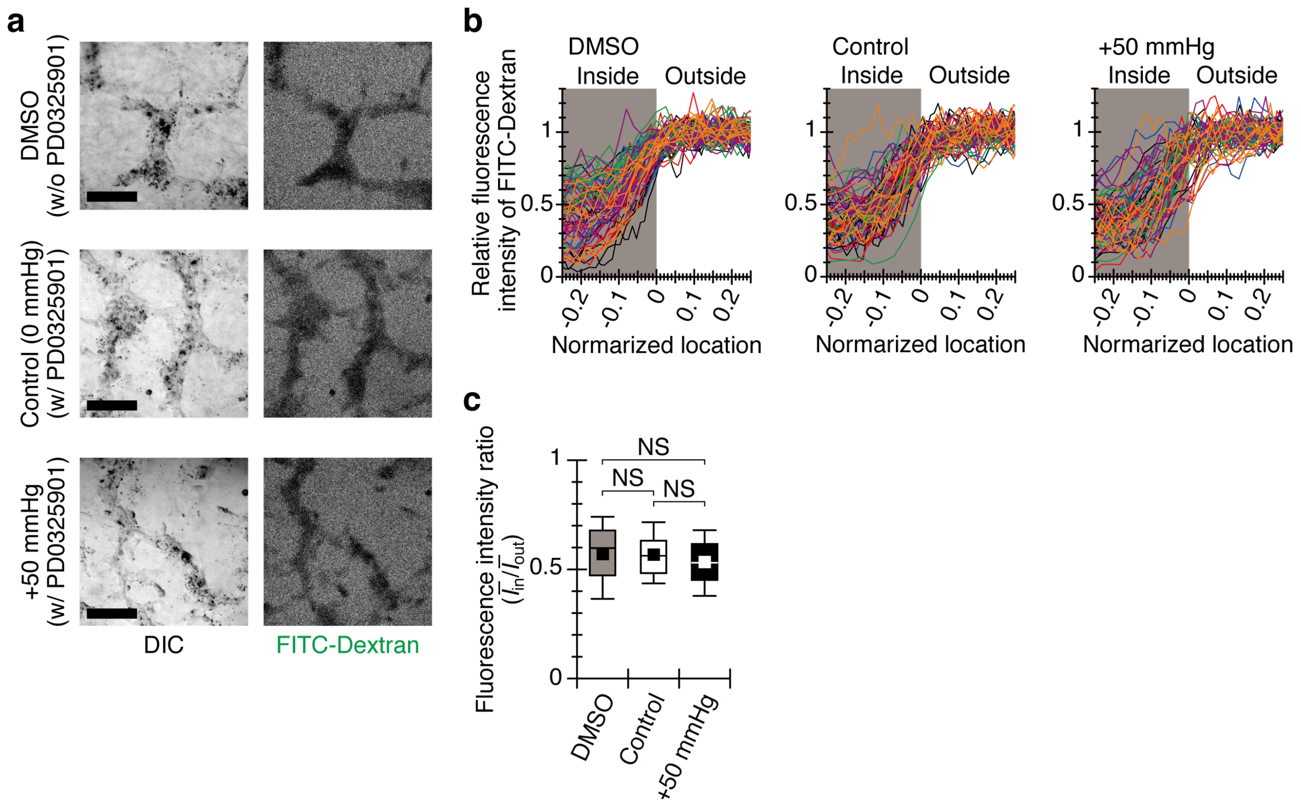

Supplementary Fig. 3.

Diffusion of 10-kDa FITC-dextran across the boundary face of a tube-like structure formed in HUVECs under the pressure condition in the presence of an MEK inhibitor (PD0325901). (**a**) Representative DIC and fluorescent images 2 h after the addition of FITC-dextran. Scale bars, 100 μm. (**b**) Line profiles of the normalized fluorescence intensity in 62 (DMSO) or 60 (control and pressure conditions) locations across the boundary face of the tube-like structures from six experiments. (**c**) Ratio of fluorescence intensity between the inside and the outside of tube-like structures shown as box-and-whisker plots (n = 62 [DMSO] or 60 [control and pressure conditions] locations). Whiskers represent the 10th and 90th percentiles, the box represents the 25th to 75th percentiles, the central line depicts the median, and the square inside each box indicates the average value. NS: no significant difference (Tukey-Kramer test; **c**).

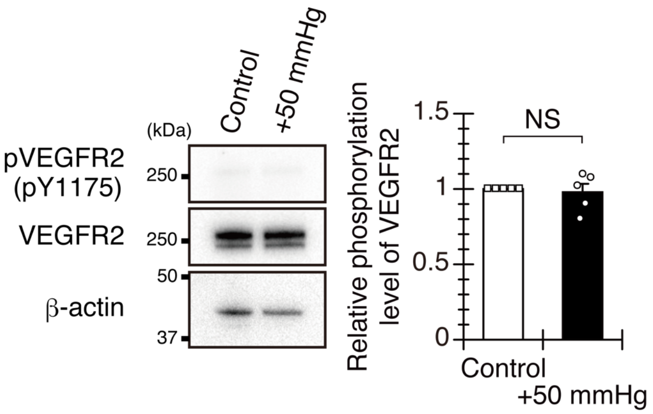

Supplementary Fig. 4.

Tyrosine residue 1175 (Y1175) of VEGFR2 is not phosphorylated after hydrostatic pressure exposure. Although VEGFR2 residue Y1175 plays an essential role in activation of the Raf-MEK-ERK pathway [1], its phosphorylation was not observed in either control cells or cells subjected to 5-min pressure exposure (n = 5 experiments). Data shown are the mean + SEM. NS: no significant difference (Welch's t test).

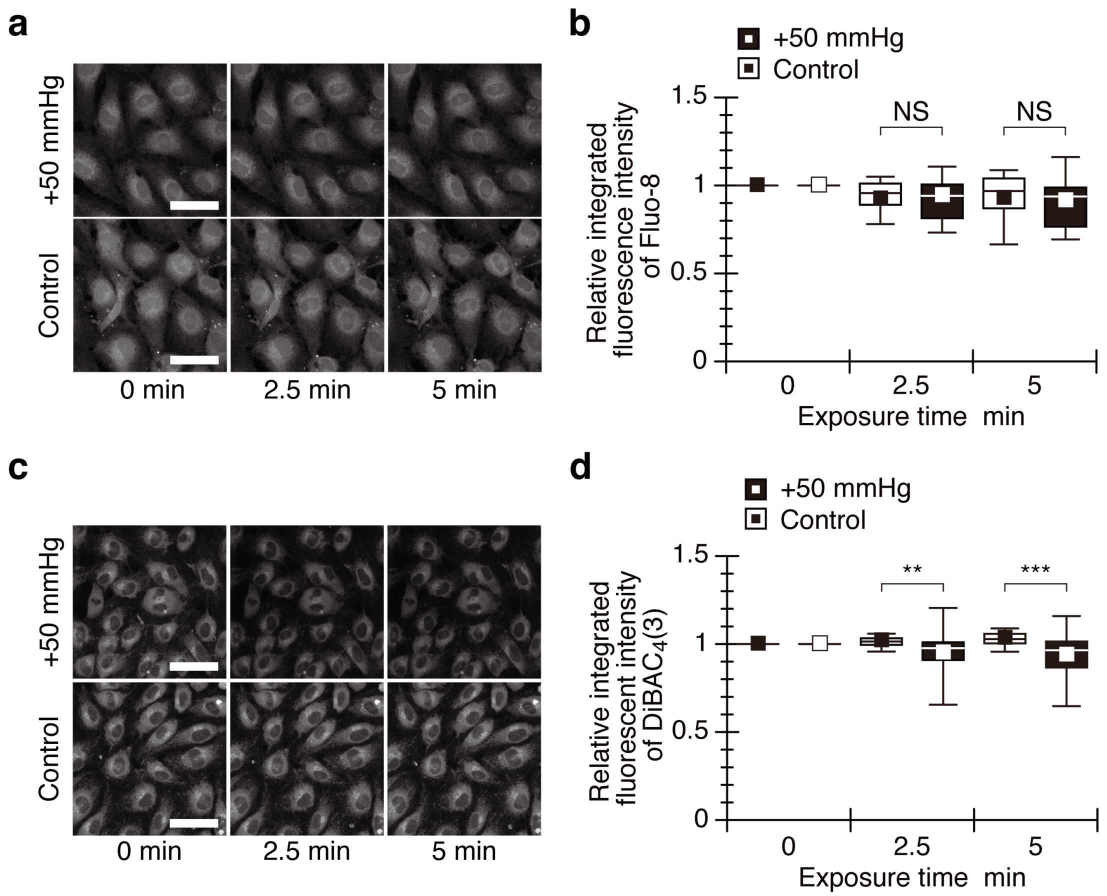

Supplementary Fig. 5.

Live cell imaging of intracellular Ca^{2+} and membrane potential in HUVECs exposed to hydrostatic pressure. (**a**) Maximum intensity projections of confocal microscopic images of intracellular Ca^{2+} (Fluo-8, AM) and (**b**) relative changes in the integrated Ca^{2+} intensity in ECs pretreated with 4 μM Fluo-8, AM for 30 min under control and pressure conditions. Each value was obtained from 50 cells in 10 independently repeated experiments (n = 50 cells). (**c**) Maximum intensity projections of confocal microscopic images of membrane potential ($DiBAC_4(3)$) and (**d**) relative change in cellular membrane potential in ECs pretreated with 4 μM $DiBAC_4(3)$ for 30 min under control and pressure conditions (*right*). Each value was obtained from 50 cells in 10 independently repeated experiments (n = 50 cells). Whiskers represent the 10th and 90th percentiles, the box represents the 25th to 75th percentiles, the central line depicts the median, and the square inside each box indicates the average value. $^{**}p < 0.05$, $^{***}p < 0.01$, NS: no significant difference (Welch's t test; **a**, **b**).

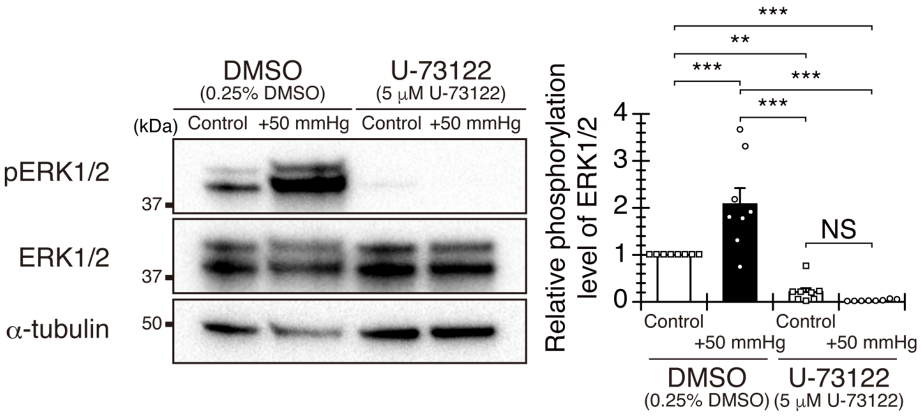

Supplementary Fig. 6.

ERK1/2 activation in HUVECs after a 5-min exposure to hydrostatic pressure in the presence of a PLC inhibitor (U-73122). No activation of ERK was observed under either the control or pressure conditions in the presence of the inhibitor (n = 8 experiments). Data shown are the mean + SEM. **$p < 0.05$, ***$p < 0.01$, NS: no significant difference (Tukey-Kramer test).

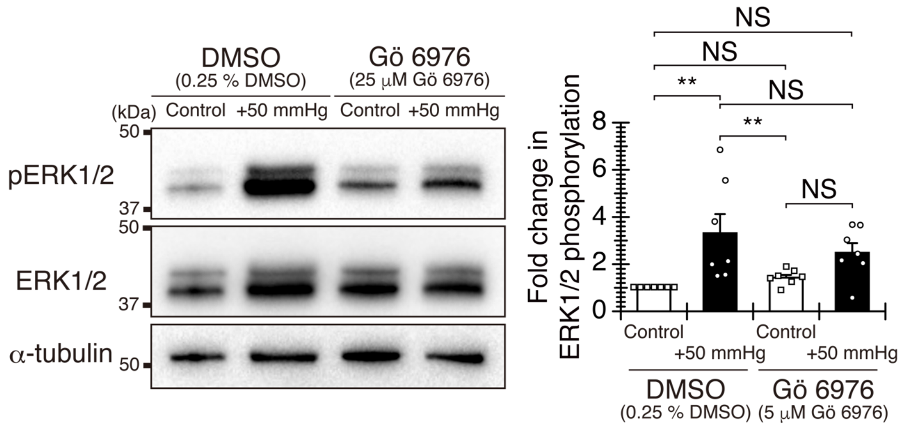

Supplementary Fig. 7.

ERK1/2 activation in HUVECs after a 5-min exposure to hydrostatic pressure in the presence of a conventional PKC inhibitor (Gö 6976). No significant difference between the control and pressure conditions was observed in the presence of the inhibitor. However, the inhibitor for the conventional PKCs did not decrease ERK1/2 activation in HUVECs after the exposure to pressure ($n = 7$ experiments). Data shown are the mean ± SEM. $^{**}p < 0.05$, NS: no significant difference (Tukey-Kramer test).

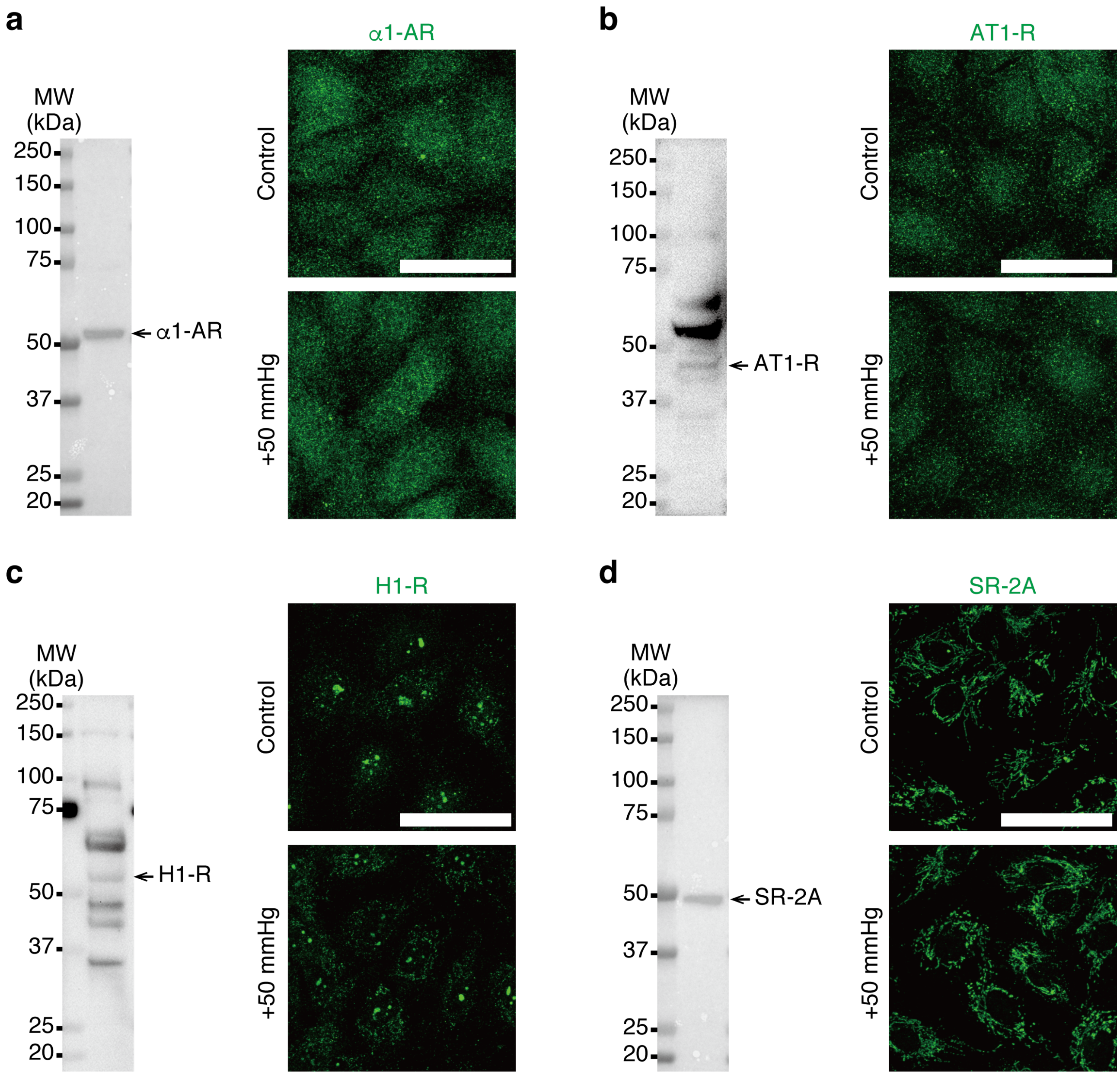

Supplementary Fig. 8.

Expression of GPCRs in HUVECs. (**a**) α1-Adrenergic receptor (α1-AR), (**b**) angiotensin II type I receptor (AT1-R), (**c**) histamine H1 receptor (H1-R), and (**d**) serotonin receptor type 2A (SR-2A). Expression of each GPCR was confirmed by immunoblotting (only control) and immunofluorescence staining. Hydrostatic pressure did not affect GPCR localization. Scale bars, 50 μm.

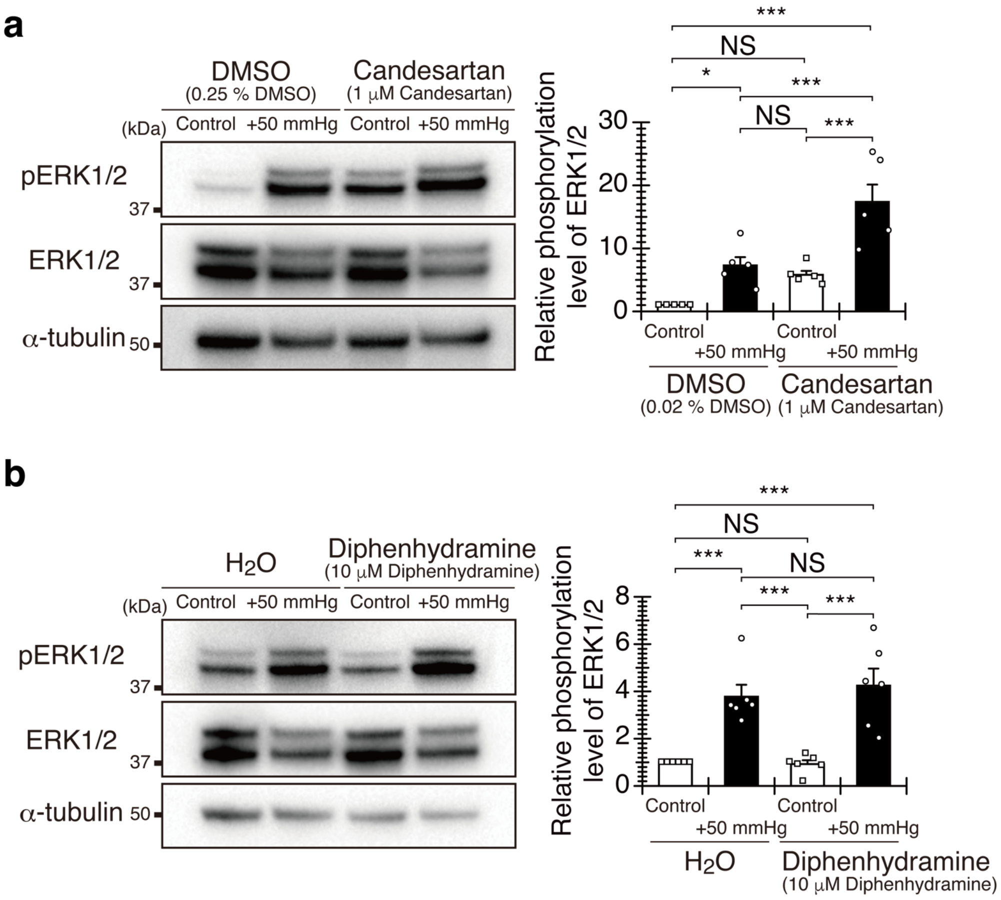

Supplementary Fig. 9.

ERK1/2 activation in HUVECs after a 5-min exposure to hydrostatic pressure in the presence of GPCR antagonists. Pressure-induced ERK1/2 activation was not inhibited even in the presence of (**a**) AT1-R antagonist (candesartan cilexetil) ($n = 5$ experiment), or (**b**) H1-R antagonist (diphenhydramine hydrochloride) ($n = 6$ experiments). All data are presented as mean ± SEM. $^{*}p < 0.1$, $^{***}p < 0.01$, NS: no significant difference (Tukey-Kramer test; **a**, **b**).

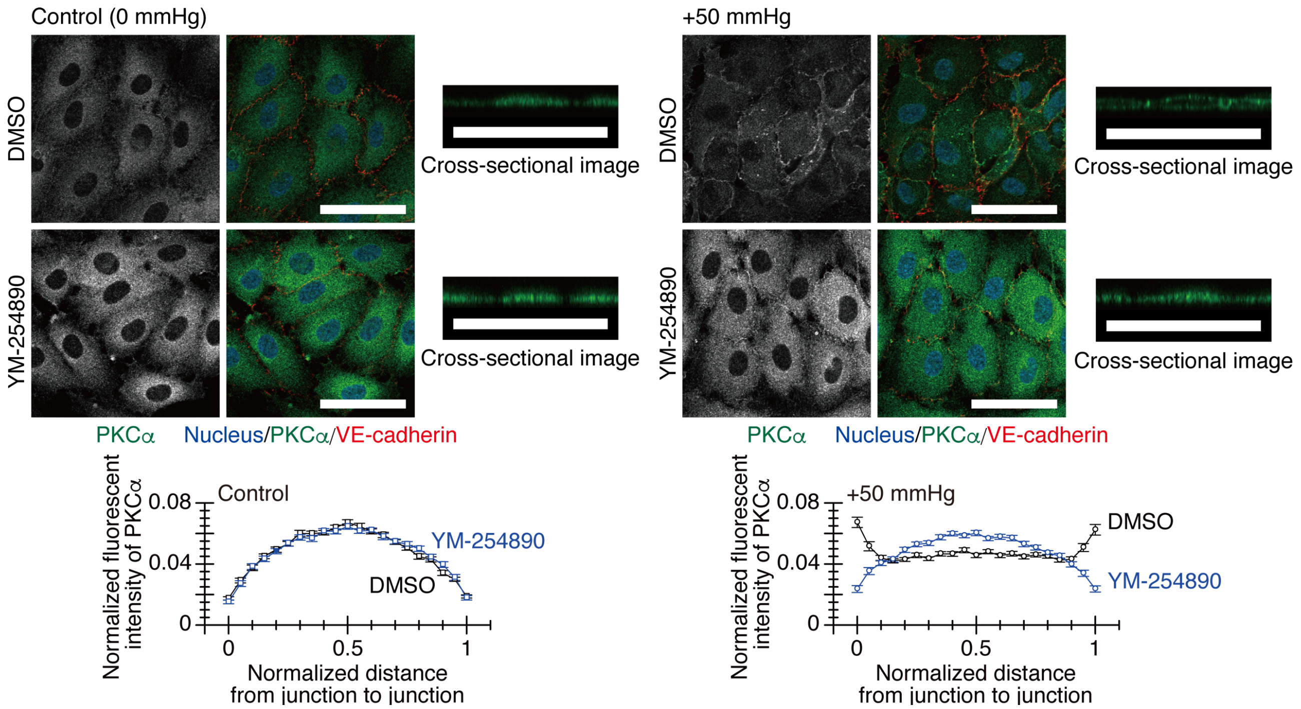

Supplementary Fig. 10.

Inhibition of G protein activation prevented pressure-induced translocation of PKC. Membrane translocation of activated PKC in HUVECs after a 5-min pressure exposure and quantified localization in 100 cells in four independently repeated experiments (n = 100 cells) with inhibition of G protein activation using YM-254890. Scale bars, 50 μm. Data shown are the mean ± SEM.

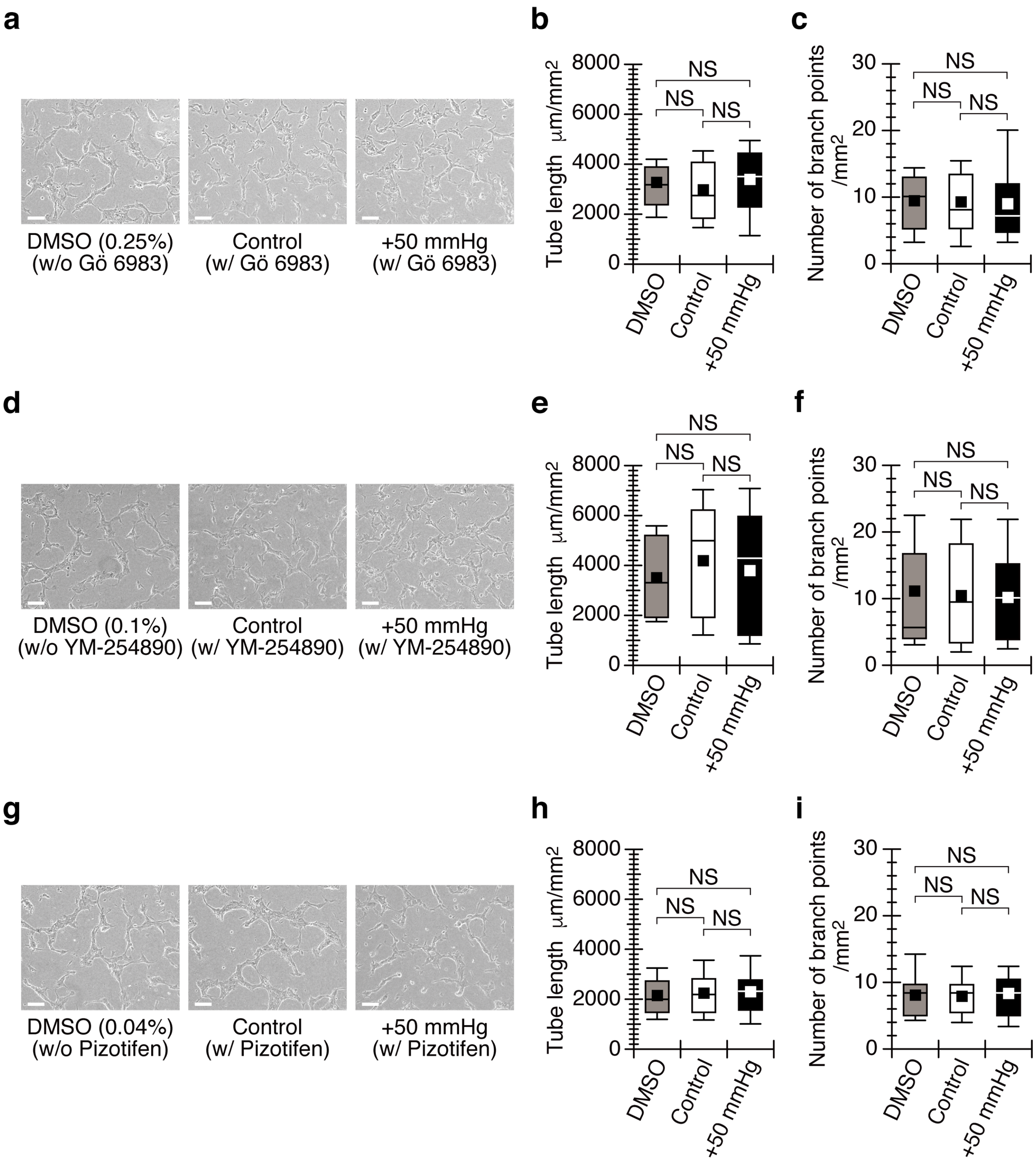

Supplementary Fig. 11.

Endothelial tube formation under the pressure condition in the presence of a (a–c) PKC inhibitor, (d–f) G protein inhibitor, or (g–i) GPCR(SR-2A) antagonist. (**a**, **d**, **g**) ECs were embedded within a collagen gel sandwich and exposed to pressure for 3 h or incubated in the control condition for 13 h. Scale bars, 100 μm. Quantified total length of tube-like structure (**b**, **e**, **h**) and number of its branch points (**c**, **f**, **i**) in a 1-mm^2 area.

Whiskers represent the 10th and 90th percentiles, the box represents the 25th to 75th percentiles, the central line depicts the median, and the square inside each box indicates the average value. Each value was obtained from 30 images, which were captured from five independently repeated experiments (*n* = 25 images). NS: no significant difference (Tukey-Kramer test; **b**, **c**, **e**, **f**, **h**, **i**).

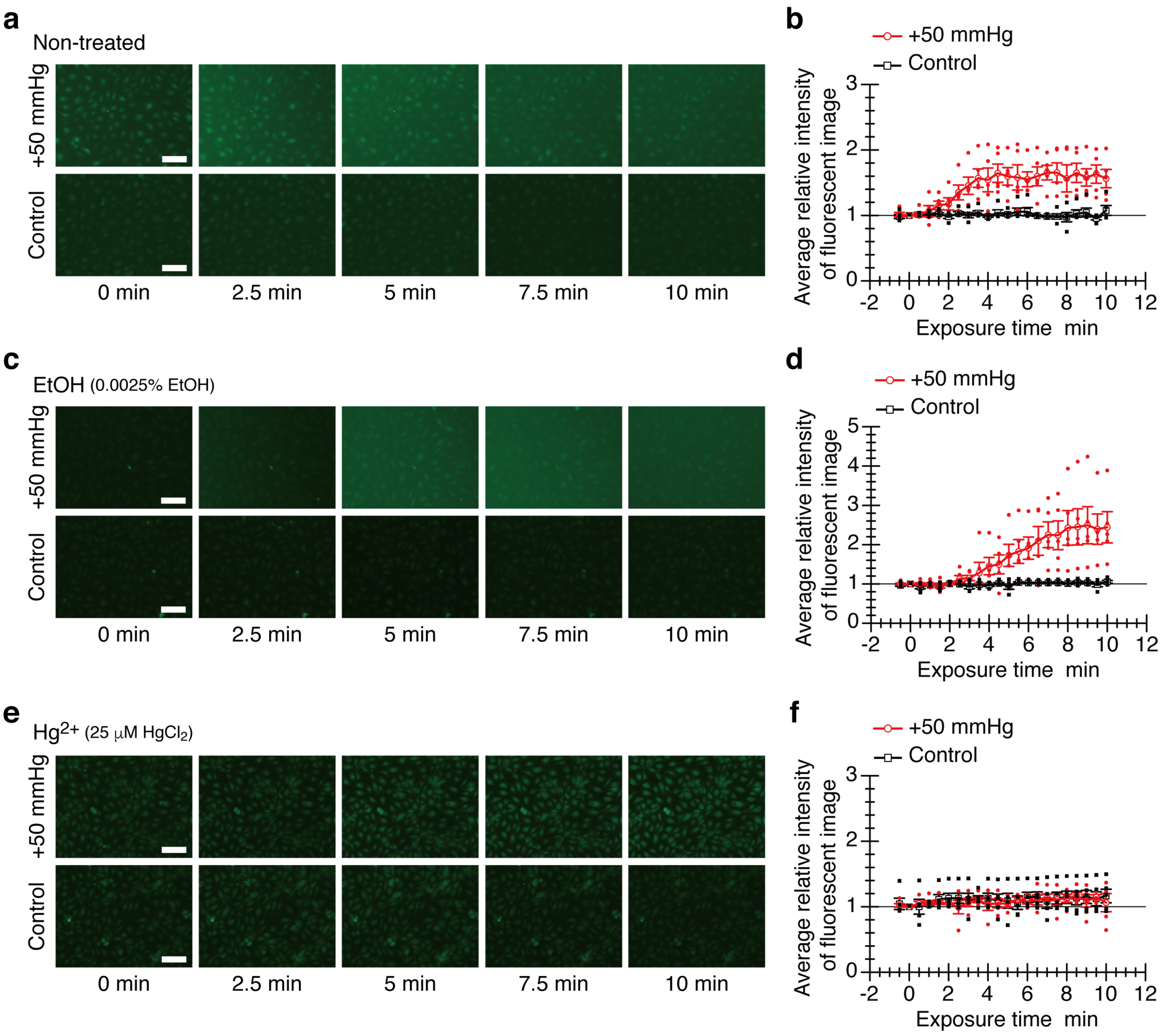

Supplementary Fig. 12.

Efflux of fluorescent Ca^{2+} indicator from cells under the pressure condition. Time sequence fluorescence images of the fluorescent Ca^{2+} indicator around HUVECs under (**a**) non-treated, (**c**) EtOH-treated, or (**e**) $HgCl_2$-treated (for inhibition of water influx and efflux) conditions. ECs were treated first with the fluorescent Ca^{2+} indicator (4 μM Fluo-8, AM) for 30 min and then washed with EM3 followed by incubation in EM3 containing 25 μM $HgCl_2$ for 15 min. Images were captured using a wide-field fluorescence microscope. (**b**, **d**, **f**) Fluorescence intensity was evaluated based on the average intensity of the images (*right*, $n = 5$ experiments). Data shown are the mean ± SEM. For the non-treated (**a**, **b**) and EtOH-treated (**c**, **d**) conditions, hydrostatic pressure induced an increase in the background fluorescence intensity but no change in the cellular Ca^{2+} ion concentration. In contrast, background fluorescence was not affected by hydrostatic pressure, and ECs exhibited no change in Ca^{2+} ion concentration under the $HgCl_2$-treated condition (**e**, **f**).

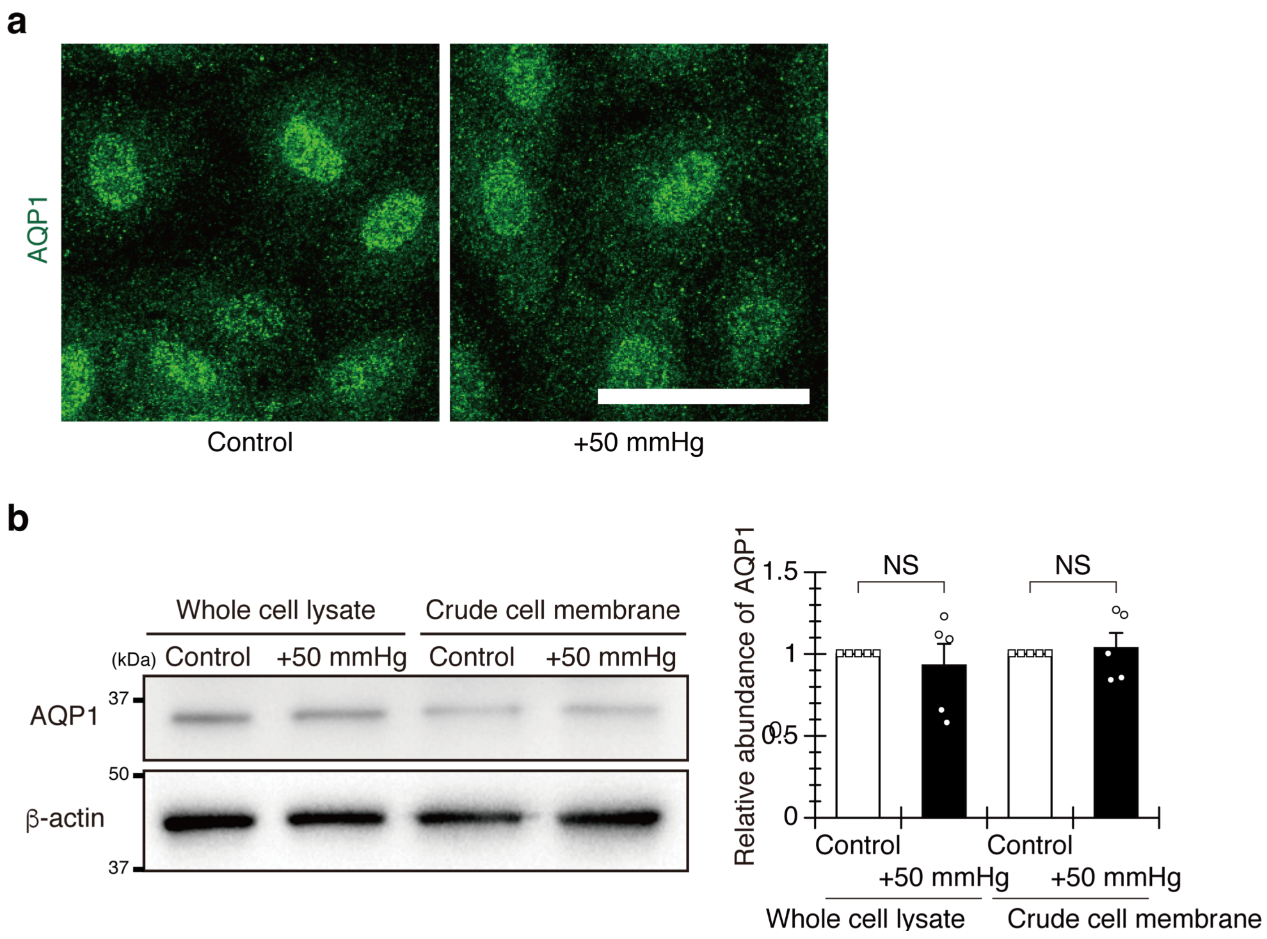

Supplementary Fig. 13.

Localization of AQP1 in HUVECs under the pressure condition. (**a**) Representative fluorescence images of AQP1 in ECs after a 5-min hydrostatic pressure exposure. Scale bar, 50 μm. (**b**) Membrane and whole AQP1 in ECs after a 5-min hydrostatic pressure exposure were analyzed by immunoblotting. The level of AQP1 expression (AQP1:β-actin) was normalized against that of the control, which was set at a value of 1 (n = 5 experiments). Data shown are the mean ± SEM. NS: no significant difference (Welch's *t* test; **b**).

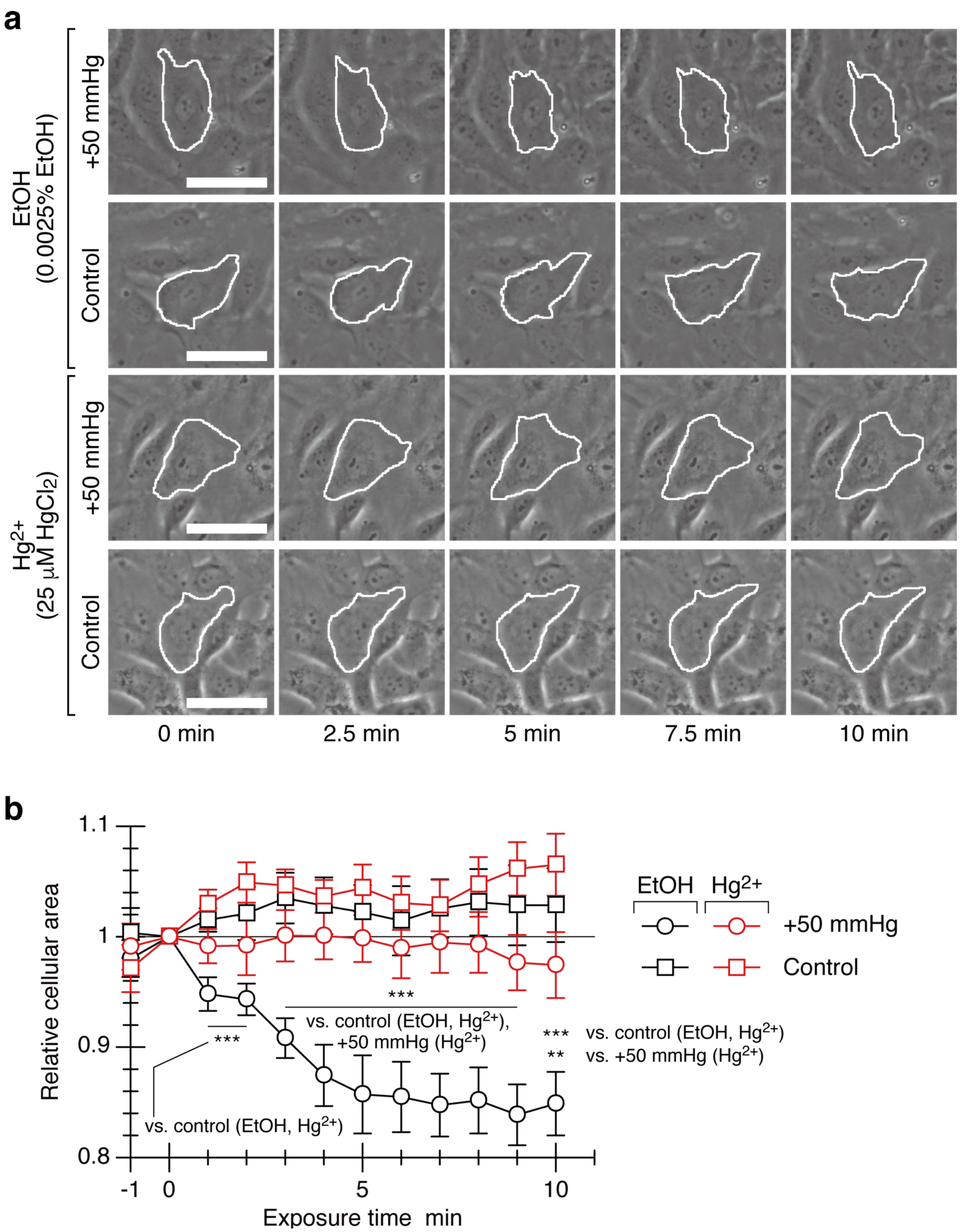

Supplementary Fig. 14.

HUVECs in which water flux was inhibited by $HgCl_2$ exhibited no contraction even if exposure to hydrostatic pressure. (**a**) Time sequence phase-contrast images depicting cell contraction and (**b**) changes in relative cell area under the pressure condition. Each value was obtained from 15 cells, which were captured in three independently repeated experiments ($n = 15$ cells). Scale bars, 50 μm. Data shown are the mean ± SEM. **$p < 0.05$, ***$p < 0.01$ (Tukey-Kramer test; **b**).

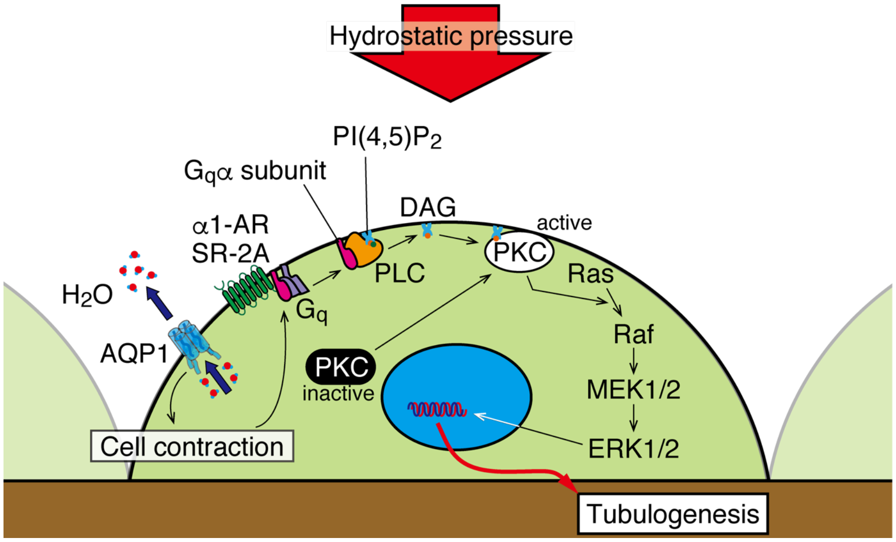

Supplementary Fig. 15.

Possible mechanism by which hydrostatic pressure promotes endothelial tubulogenesis. Hydrostatic pressure rapidly induces water efflux via AQP1, with resultant cell contraction. Specific GPCRs, α1-AR and SR-2A, are then activated, in turn activating PKC (GPCR/PLC pathway). Activated PKC activates the Ras/ERK pathway. Hydrostatic pressure promotes tube formation in ECs through this sequence of signaling pathways. An earlier study reported that AT1-R is a sensor for hydrostatic pressure in vascular smooth muscle cells [2]. However, AT1-R did not affect hydrostatic pressure sensing in HUVECs in this study, perhaps because the previous study allowed deformation of the isolated artery. The smooth muscle cells were exposed to a stretch stimulus; thus, AT1-R in the smooth muscle cells responded to the stimulus. This hypothesis is supported by reports indicating that AT1-R is activated by stretch stimuli without the involvement of angiotensin II [3].

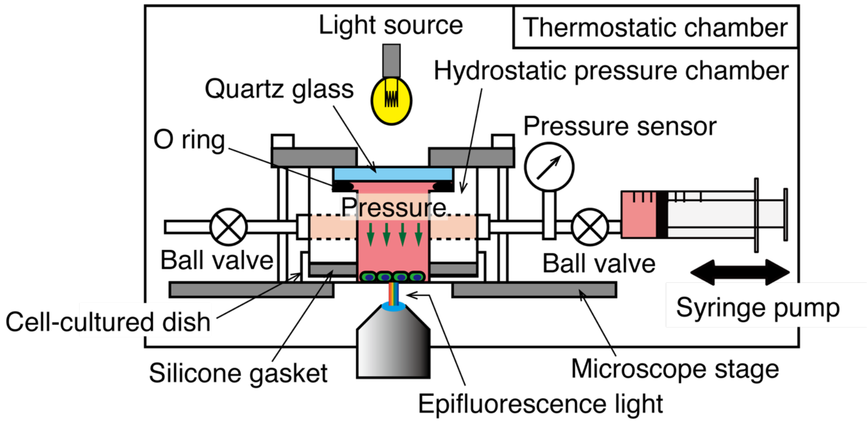

Supplementary Fig. 16.

Custom-made hydrostatic pressure microscopy system. The system consists of a cell culture dish, polycarbonate pressure chamber, silicone gasket, O-ring, quartz glass, two ball valves, a thermostatic chamber, syringe pump, and microscope. Pressure is controlled by compression and expansion of medium using the syringe pump.

Supplementary Fig. 17. Raw data for the western blots included in each figure

Fig. 1h

α-tubulin VE-cadherin ZO-1

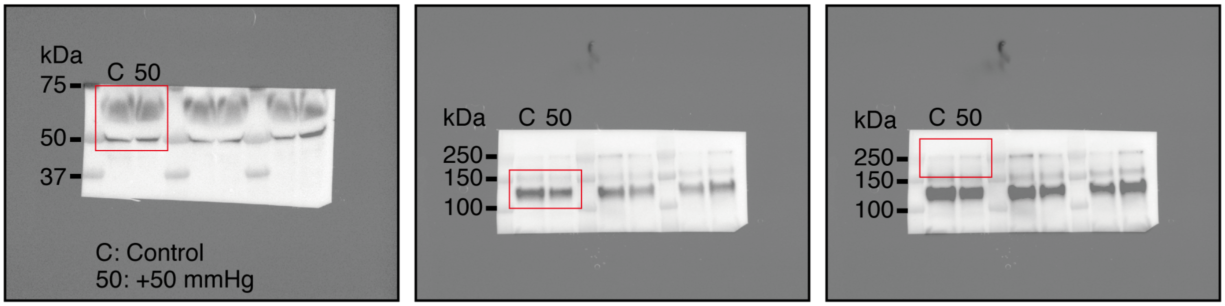

Fig. 2a

α-tubulin ERK1/2 pERK1/2

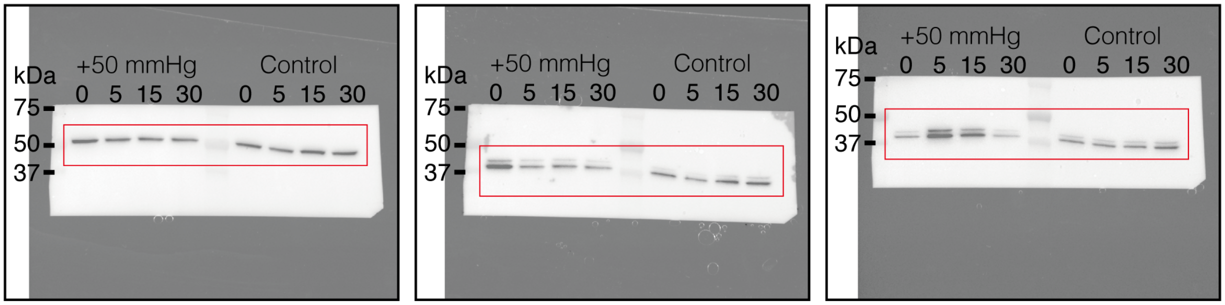

Fig. 2b

α-tubulin MEK1/2 pMEK1/2

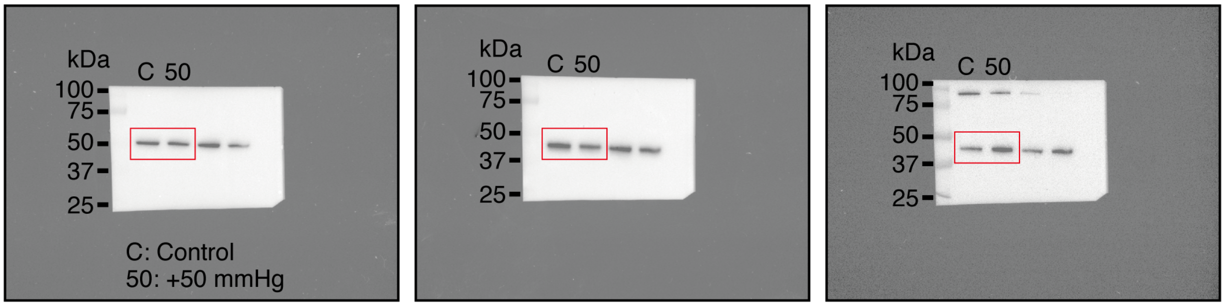

Fig. 2c

Ras

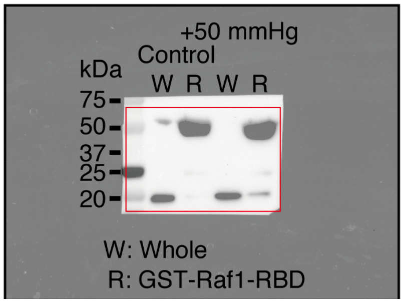

Fig. 2i

α-tubulin VE-cadherin / ZO-1

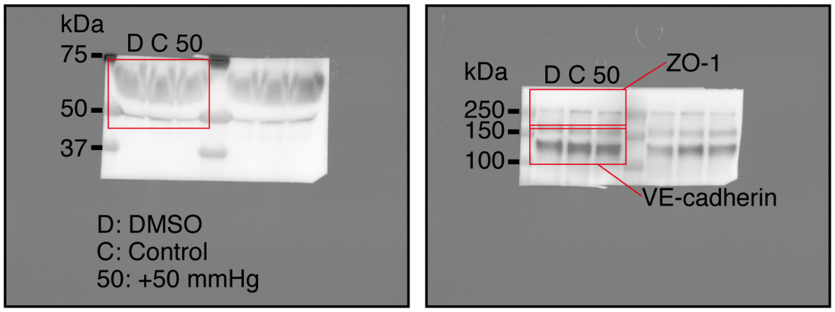

Fig. 3b

β-actin PI(4,5)P2

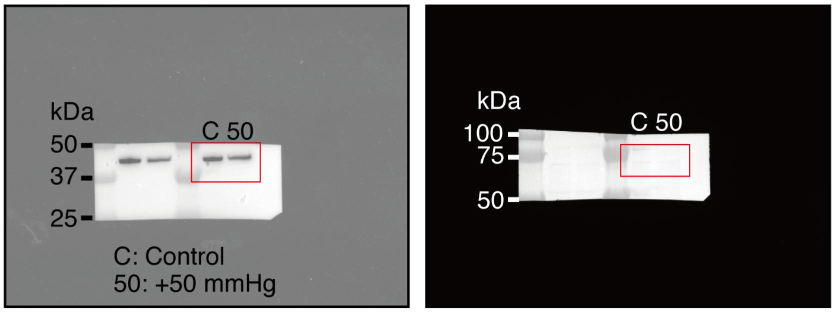

Fig. 3c

α-tubulin ERK1/2 pERK1/2

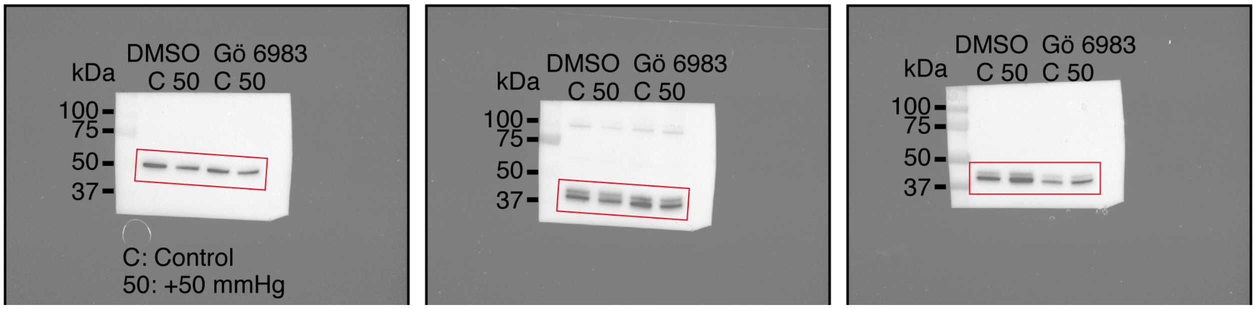

163

Fig. 3d

GAPDH (Whole cell lysate)

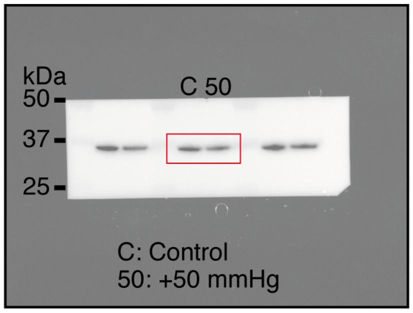

Gα q/11/14 (Whole cell lysate)

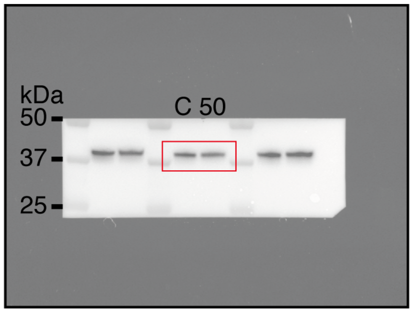

GAPDH (Cytosol fraction)

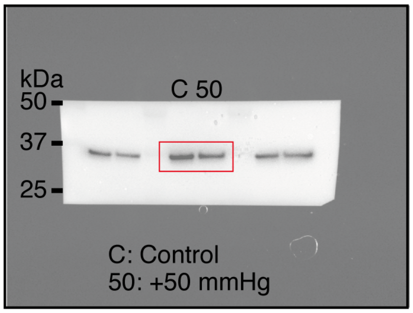

Gα q/11/14 (Cytosol fraction)

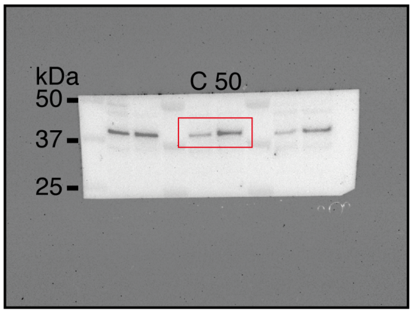

Fig. 3e

α-tubulin

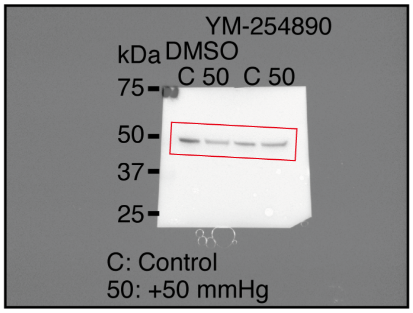

ERK1/2

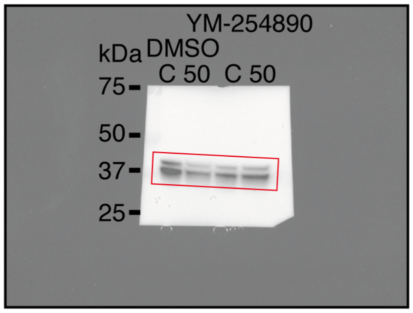

pERK1/2

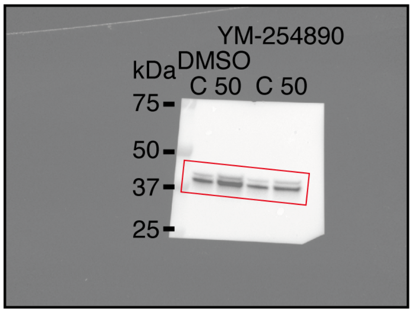

Fig. 3f

α-tubulin

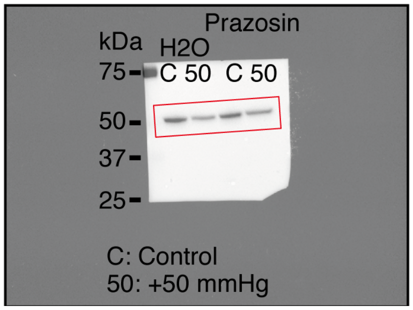

ERK1/2

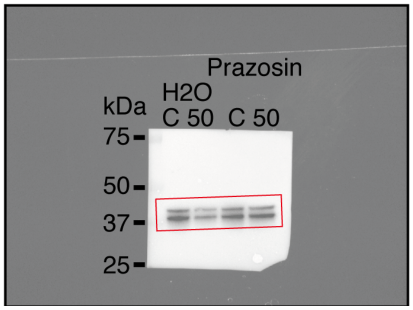

pERK1/2

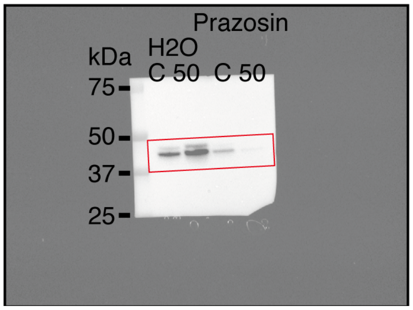

Fig. 3g

α-tubulin ERK1/2 pERK1/2

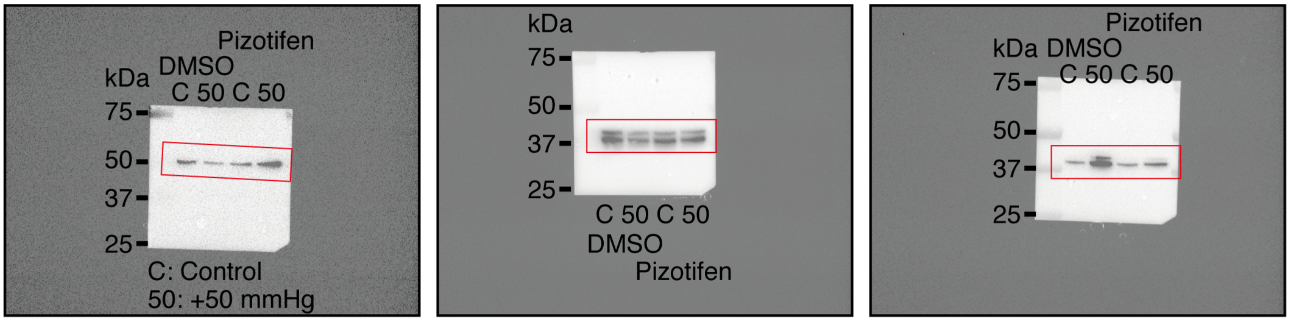

Fig. 4c

α-tubulin ERK1/2 pERK1/2

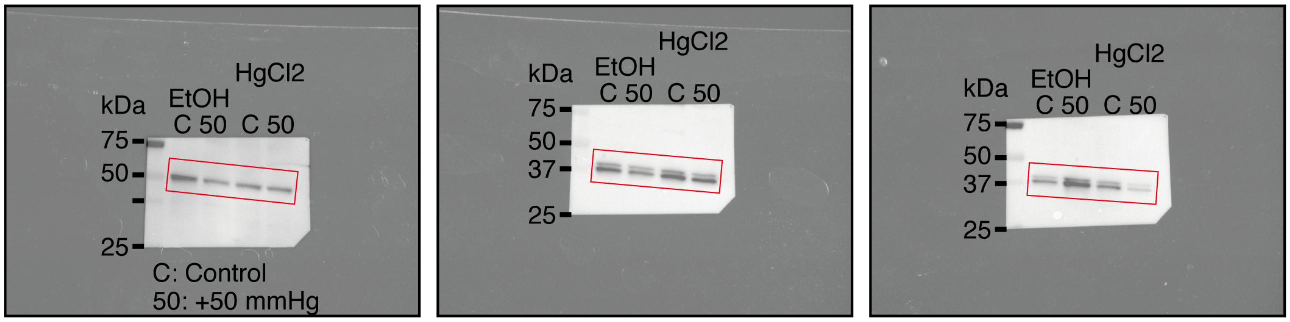

Fig. 4d

α-tubulin MEK1/2 pMEK1/2

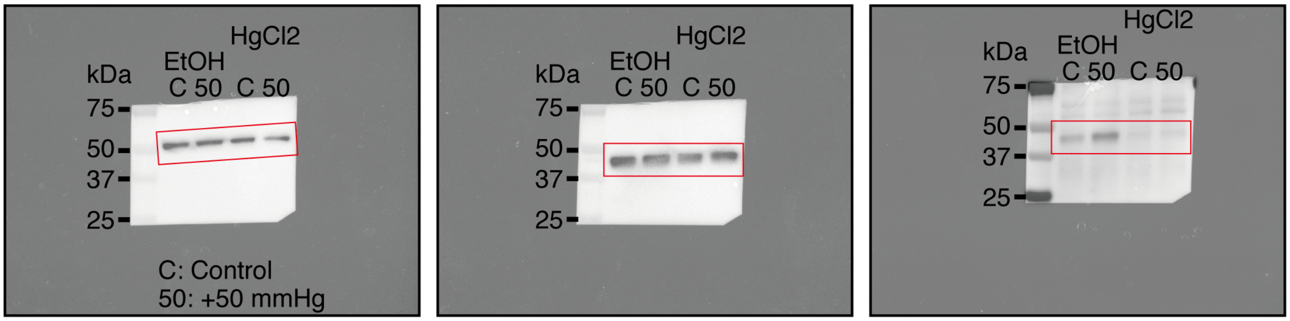

Fig. 4e

Ras (EtOH) Ras (HgCl2)

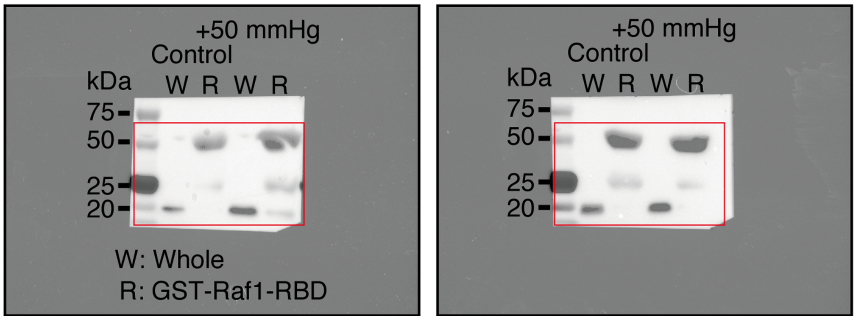

Supplementary Fig. 5

β-actin

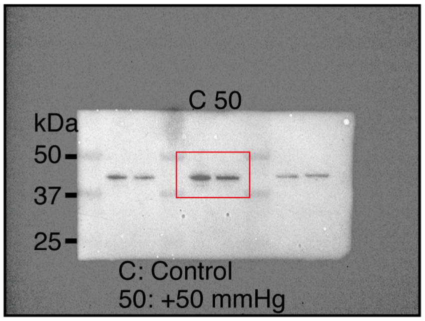

VEGFR2

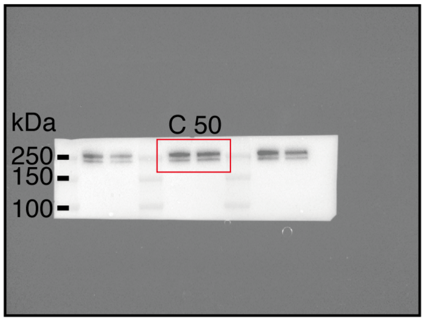

pVEGFR2 (pY1175)

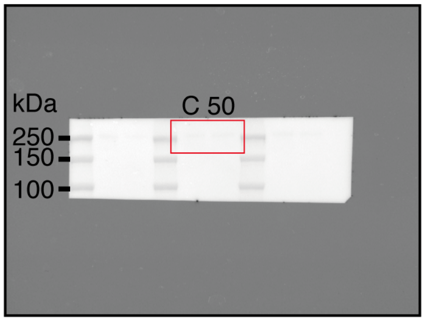

Supplementary Fig. 7

α-tubulin

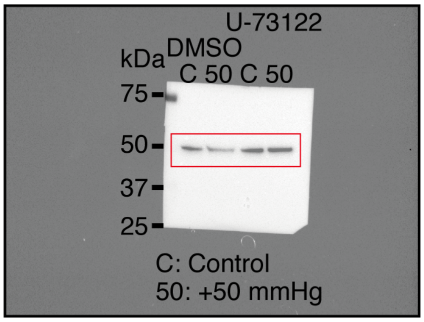

ERK1/2

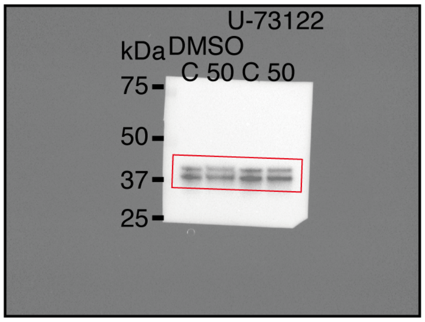

pERK1/2

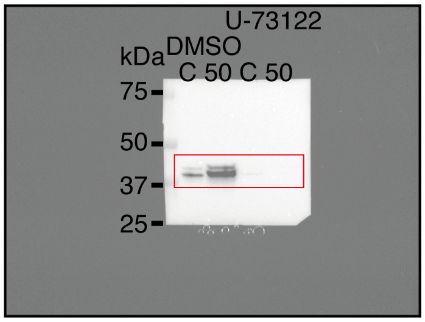

Supplementary Fig. 8

α-tubulin

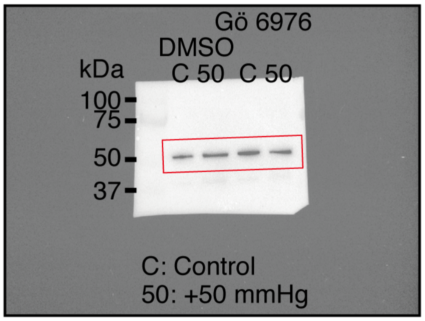

ERK1/2

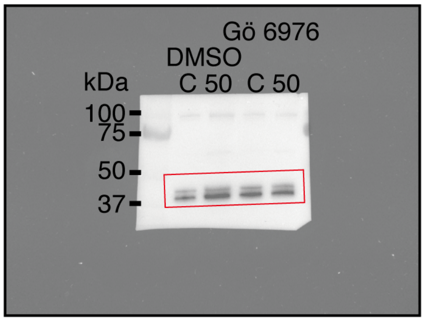

pERK1/2

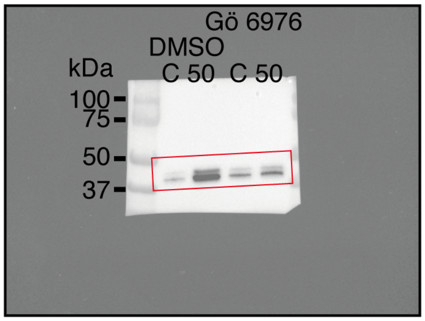

Supplementary Fig. 9a, b

α1-AR

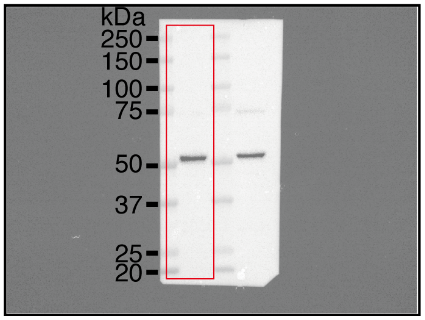

AT1-R

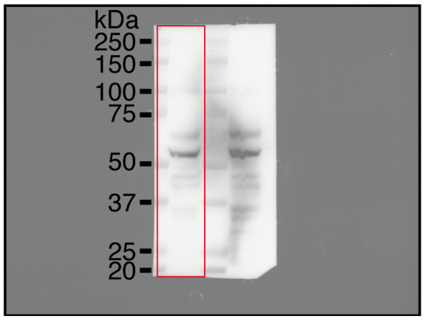

Supplementary Fig. 9c, d

H1-R

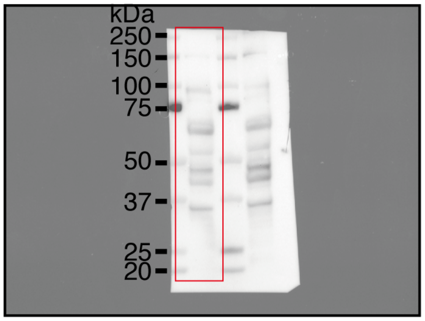

SR-2A

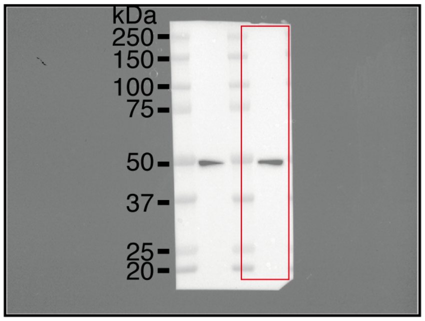

Supplementary Fig. 10a

α-tubulin

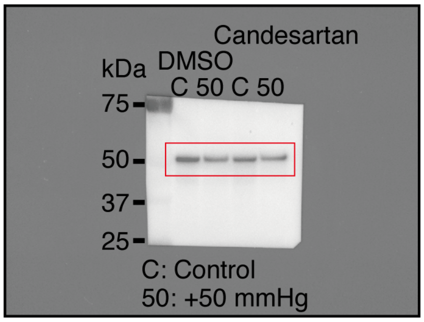

ERK1/2

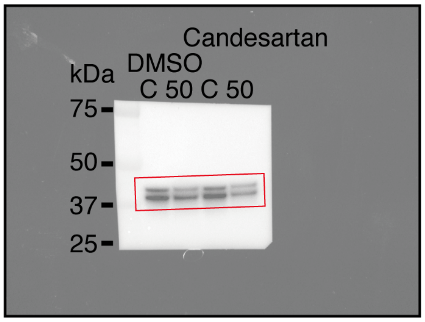

pERK1/2

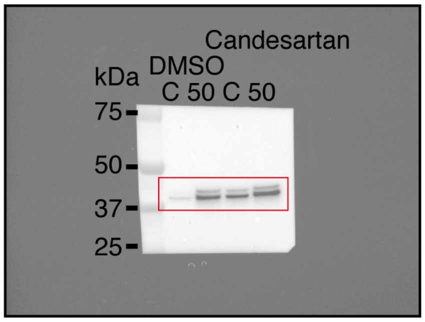

Supplementary Fig. 10b

α-tubulin

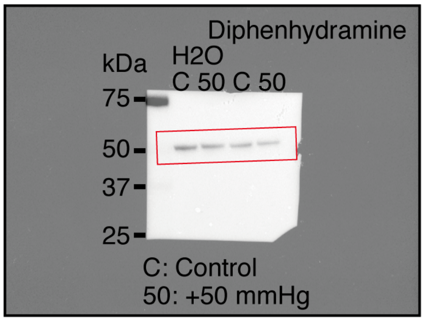

ERK1/2

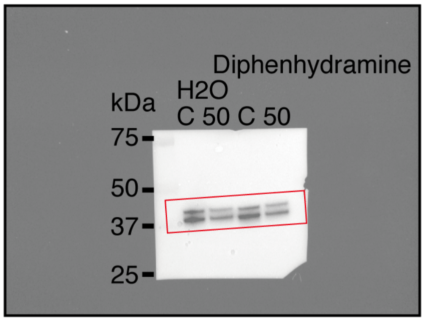

pERK1/2

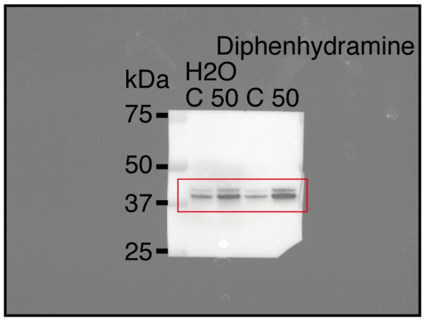

Supplementary Fig. 14b

β-actin

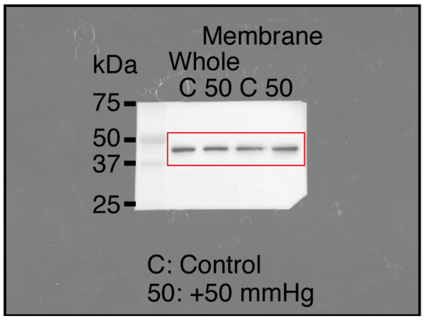

AQP1

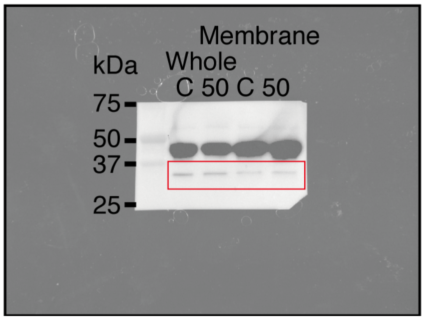

Supplementary Table 1.

List of inhibitors and antagonists used in this study

Substance	Supplier	Catalog #	Target	Incubation time before experiment	Final concentration (Solvent)
**PD0325901**	Sigma-Aldrich	PZ0162	MEK1/2 (ERK activation)	30 min	1 μM (DMSO)
**U-73122 hydrate**	Sigma-Aldrich	U6756	PLC	30 min	5 μM (DMSO)
**Gö 6983**	Sigma-Aldrich	G1918	PKC	30 min	5 μM (DMSO)
**Gö 6976**	Abcam	ab141413	Ca^{2+}-dependent PKC isoforms	30 min	5 μM (DMSO)
**YM-254890**	Wako Pure Chemical Industries	257-00631	$G\alpha_{q/11/14}$	10 min	1 μM (DMSO)
**Prazosin hydrochloride**	Sigma-Aldrich	P7791	α1-Adrenergic receptor	2 hours	50 μM (H_2O)
**Candesartan cilexetil**	Tokyo Chemical Industry	C2635	Angiotensin II type 1 receptor	3 hours	1 μM (DMSO)
**Diphenhydramine hydrochloride**	Sigma-Aldrich	D3630	Histamine H1 Receptor	2 hours	10 μM (H_2O)
**Pizotifen**	Tokyo Chemical Industry	P2344	Serotonin Receptor-2A	2 hours	10 μM (DMSO)
**Mercury (II) Chloride ($HgCl_2$)**	Wako Pure Chemical Industries	139-09362	Aquaporin 1	15 min	25 μM (EtOH)

Supplementary Table 2.

List of primary antibodies used in this study

Substance	Host species	Supplier	Catalog #	Dilution (Application)
**Anti-Cyclin D1**	Rabbit	Santa Cruz Biotechnology	sc-753	1:100 (IF)
**Anti-alpha Tubulin**	Mouse	Abcam	ab7291	1:10000 (WB)
**Anti-ERK1 + ERK2**	Rabbit	Abcam	ab17942	1:1000 (WB)
**Anti-ERK1/2**	Rabbit	Cell Signaling Technology	4695	1:1000 (WB)
**Anti-phospho-ERK1(pT202/pY204)+ ERK2(pT185/pY187)**	Mouse	Abcam	ab50011	1:100 (IF) 1:10000 (WB)
**Anti-MEK1 + MEK2**	Rabbit	Abcam	ab178876	1:5000 (WB)
**Anti-MEK1 + MEK2 (phospho S218 + S222)**	Rabbit	Abcam	ab194754	1:1000 (WB)
**Anti-β-Actin**	Mouse	Santa Cruz Biotechnology	sc-47778	1:1000 (WB)
**Anti-VEGF Receptor 2**	Rabbit	Cell Signaling Technology	2479	1:1000 (WB)
**Anti-phospho-VEGF Receptor 2**	Rabbit	Cell Signaling Technology	2478	1:1000 (WB)
**Anti-PKC alpha**	Rabbit	Abcam	ab32376	1:200 (IF)
**Anti-ZO-1**	Mouse	Invitrogen	33-9100	1:100 (IF) 1:500 (WB)
**Anti-VE-cadherin**	Mouse	Santa Cruz Biotechnology	sc-9989	1:100 (IF)
**Anti-VE-cadherin**	Rabbit	Invitrogen	PA5-17501	1:100 (IF) 1:1000 (WB)
**Anti-PIP_2**	Mouse	Santa Cruz Biotechnology	sc-53412	1:1000 (WB)
**Anti-GAPDH**	Mouse	Abcam	ab8245	1:10000 (WB)
**Anti-$G\alpha_{q/11/14}$**	Mouse	Santa Cruz Biotechnology	sc-365906	1:200 (WB)
**Anti-alpha1 Adrenegic Receptor**	Rabbit	Abcam	ab3462	1:200 (IF) 1:400(WB)
**Anti-AT_1 (Angiotensin II type 1 receptor)**	Mouse	Santa Cruz Biotechnology	sc-57036	1:100 (IF) 1:200 (WB)

**Anti-Histamine H1 Receptor**	Mouse	Santa Cruz Biotechnology	sc-374621	1:100 (IF)<br>1:500 (WB)
**Anti-SR-2A (Serotonin Receptor-2A)**	Mouse	Santa Cruz Biotechnology	sc-166775	1:100 (IF)<br>1:1000(WB)
**Anti-Aquaporin 1**	Rabbit	Abcam	ab125041	1:200 (IF)<br>1:1000 (WB)

Supplementary Table 3.

List of secondary antibodies used in this study

Substance	Conjugated label	Supplier	Catalog #	Dilution (Application)
**Goat anti-mouse IgG (H+L)**	Alexa Fluor 488	Invitrogen	A11001	1:200 (IF)
**Goat anti-mouse IgG (H+L)**	Alexa Fluor 594	Invitrogen	A11005	1:200 (IF)
**Goat anti-mouse IgM (Heavy chain)**	Alexa Fluor 488	Invitrogen	A21042	1:200 (IF)
**Goat anti-rabbit IgG (H+L)**	Alexa Fluor 488	Invitrogen	A11008	1:200 (IF)
**Goat anti-rabbit IgG (H+L)**	Alexa Fluor 594	Invitrogen	A11012	1:200 (IF)
**Anti-mouse IgG**	HRP	Cell Signaling Technology	7076	1:2000~1:5000 (WB)
**Goat anti-mouse IgM**	AP	Santa Cruz Biotechnology	sc-2978	1:1000 (WB)
**Anti-rabbit IgG**	HRP	Cell Signaling Technology	7074	1:2000~1:5000 (WB)

Supplementary Reference

1. Takahashi, H. & Shibuya, M. The vascular endothelial growth factor (VEGF)/VEGF receptor system and its role under physiological and pathological conditions. *Clin Sci.* **109**, 227–241 (2005).
2. Pires, P. W. *et al.* The angiotensin II receptor type 1b is the primary sensor of intraluminal pressure in cerebral artery smooth muscle cells. *J. Physiol.* **595**, 4735–4753 (2017).
3. Zou, Y. *et al.* Mechanical stress activates angiotensin II type 1 receptor without the involvement of angiotensin II. *Nat. Cell Biol.* **6**, 499–506 (2004).
