# Supplementary material for: Hydrostatic pressure promotes endothelial tube formation through aquaporin 1 and Ras-ERK signaling
Source: Commun Biol. 2020 Apr 2;3:152. doi: 10.1038/s42003-020-0881-9 (PMC7118103; doi:10.1038/s42003-020-0881-9)
Supplement: Supplementary file 2 — Description of Additional Supplementary Files [file 42003_2020_881_MOESM2_ESM.pdf]

Description of Additional Supplementary Files

File Name: Supplementary Movie 1
Description: Morphological dynamics of HUVECs under the control condition, which were captured with a phase contrast microscopy. Movie shows 10-min change at the frame interval of 15 sec. Scale bar = 50 μm.

File Name: Supplementary Movie 2
Description: Morphological dynamics of HUVECs under the pressured condition. Movie starts 1 min before applying pressure to cells and lasts 10 min. Frame interval is 15 sec. Scale bar = 50 μm.

File Name: Supplementary Movie 3
Description: Morphological dynamics of HUVECs under the control condition in the presence of EtOH, which was used as a solvent for $HgCl_2$. Movie shows 10-min change at the frame interval of 15 sec. Scale bar = 50 μm.

File Name: Supplementary Movie 4
Description: Morphological dynamics of HUVECs under the pressured condition in the presence of EtOH, which was used as a solvent for $HgCl_2$. Movie starts 1 min before applying pressure to cells and lasts 10 min. Frame interval is 15 sec. Scale bar = 50 μm.

File Name: Supplementary Movie 5
Description: Morphological dynamics of HUVECs, in which water flux was inhibited with $HgCl_2$, under the control condition. Movie shows 10-min change at the frame interval of 15 sec. Scale bar = 50 μm.

File Name: Supplementary Movie 6
Description: Morphological dynamics of HUVECs, in which water flux was inhibited with $HgCl_2$, under the pressured condition. Movie starts 1 min before applying pressure to cells and lasts 10 min. Frame interval is 15 sec. Scale bar = 50 μm.

File Name: Supplementary Data 1

Description: Table containing all source data underlying the graphs presented in the main and supplementary figures.

File Name: Supplementary Data 2

Description: The exact *p*-values and the effect size for all statistically tested data.
