# Supplementary material for: Hydrostatic pressure promotes endothelial tube formation through aquaporin 1 and Ras-ERK signaling
Source: Commun Biol. 2020 Apr 2;3:152. doi: 10.1038/s42003-020-0881-9 (PMC7118103; doi:10.1038/s42003-020-0881-9)
Supplement: Supplementary file 11 — Reporting Summary [file 42003_2020_881_MOESM11_ESM.pdf]

nature research

Corresponding author(s): Daisuke Yoshino

Last updated by author(s): Feb 24, 2020

Reporting Summary

Nature Research wishes to improve the reproducibility of the work that we publish. This form provides structure for consistency and transparency in reporting. For further information on Nature Research policies, see Authors & Referees and the Editorial Policy Checklist.

Statistics

For all statistical analyses, confirm that the following items are present in the figure legend, table legend, main text, or Methods section.

n/a	Confirmed	
[ ]	[x]	The exact sample size (*n*) for each experimental group/condition, given as a discrete number and unit of measurement
[ ]	[x]	A statement on whether measurements were taken from distinct samples or whether the same sample was measured repeatedly
[ ]	[x]	The statistical test(s) used AND whether they are one- or two-sided<br>*Only common tests should be described solely by name; describe more complex techniques in the Methods section.*
[x]	[ ]	A description of all covariates tested
[ ]	[x]	A description of any assumptions or corrections, such as tests of normality and adjustment for multiple comparisons
[ ]	[x]	A full description of the statistical parameters including central tendency (e.g. means) or other basic estimates (e.g. regression coefficient) AND variation (e.g. standard deviation) or associated estimates of uncertainty (e.g. confidence intervals)
[ ]	[x]	For null hypothesis testing, the test statistic (e.g. *F*, *t*, *r*) with confidence intervals, effect sizes, degrees of freedom and *P* value noted<br>*Give P values as exact values whenever suitable.*
[x]	[ ]	For Bayesian analysis, information on the choice of priors and Markov chain Monte Carlo settings
[x]	[ ]	For hierarchical and complex designs, identification of the appropriate level for tests and full reporting of outcomes
[ ]	[x]	Estimates of effect sizes (e.g. Cohen's *d*, Pearson's *r*), indicating how they were calculated

Our web collection on statistics for biologists contains articles on many of the points above.

Software and code

Policy information about availability of computer code

Data collection	- Microscopy images were collected using NIS elements software (Nikon), AxioVision software and ZEN imaging software (Zeiss), EVOS FL Cell Imaging System (Thermo Fisher Scientific), and EVOS FL Auto2 Imaging System (Thermo Fisher Scientific).<br>- Flow cytometry data was collected using InCyte software on a Guava easyCyteTM 6HT flow cytometer (Merck Millipore).<br>- Immunoblotting data was collected using Image Lab software on a ChemiDoc XRS+ Imager (Bio-Rad).
Data analysis	- Microsoft Excel or R software was used for statistical analysis (the two-sided Welch's t-test for comparisons of two groups).<br>- R software was used for statistical analysis (the Tukey-Kramer test for multiple comparisons).<br>- ImageJ software (US National Institute for Health) was used for tube formation assay, evaluation for maturation of tube-like structures, evaluation for nuclear/cytoplasm ratios of cyclin D1 and activated ERK, evaluation of cell morphology, and assessment of Ca2+ ion concentration and cellular membrane potential.<br>- ZEN imaging software (Zeiss) was used for measurement of line profiles of fluorescent intensity of PKC alpha.<br>- Image Lab (Bio-Rad) was used to determine the density of protein bands on immunoblots.

For manuscripts utilizing custom algorithms or software that are central to the research but not yet described in published literature, software must be made available to editors/reviewers. We strongly encourage code deposition in a community repository (e.g. GitHub). See the Nature Research guidelines for submitting code & software for further information.

Data

Policy information about availability of data

All manuscripts must include a data availability statement. This statement should provide the following information, where applicable:

- Accession codes, unique identifiers, or web links for publicly available datasets
- A list of figures that have associated raw data
- A description of any restrictions on data availability

All data supporting the findings of this study are available from the corresponding author on reasonable request.

Field-specific reporting

Please select the one below that is the best fit for your research. If you are not sure, read the appropriate sections before making your selection.

☒ Life sciences ☐ Behavioural & social sciences ☐ Ecological, evolutionary & environmental sciences

For a reference copy of the document with all sections, see nature.com/documents/nr-reporting-summary-flat.pdf

Life sciences study design

All studies must disclose on these points even when the disclosure is negative.

Sample size	We used no statistical method for determination of sample size. The sample size for each experiment was sufficient to yield clear statistical significance.
Data exclusions	No data was excluded from the analyses.
Replication	All data in this study was based on at least 3 independent experiments. All attempts at replication were successful. All the reagents, chemicals, and antibodies used in this study are indicated, and the methods, including a custom-made system for in this study, are described in detail.
Randomization	Each experiment was conducted by using randomly selected samples cultured in independent repeats. Captured images for measurements of cell morphology and fluorescent intensity of the target proteins were randomly captured from samples cultured in independent repeats.
Blinding	Measurement of line profiles of fluorescent intensity of FITC-dextran or PKC alpha, evaluation of cell morphology, and assessment of Ca2+ ion concentration and cellular membrane potential were manual, and therefore computationally blinded. All other analyses were automatically performed by softwares, and therefore non-blinded.

Reporting for specific materials, systems and methods

We require information from authors about some types of materials, experimental systems and methods used in many studies. Here, indicate whether each material, system or method listed is relevant to your study. If you are not sure if a list item applies to your research, read the appropriate section before selecting a response.

Materials & experimental systems

n/a	Involved in the study	
☐	☒	Antibodies
☐	☒	Eukaryotic cell lines
☒	☐	Palaeontology
☒	☐	Animals and other organisms
☒	☐	Human research participants
☒	☐	Clinical data

Methods

n/a	Involved in the study	
☒	☐	ChIP-seq
☐	☒	Flow cytometry
☒	☐	MRI-based neuroimaging

Antibodies

Antibodies used	Detailed information about primary and secondary antibodies used in this study are described in Supplementary Table 2 and 3.
Validation	Antibodies were used for applications that were validated by the manufacturers.

Eukaryotic cell lines

Policy information about cell lines

Cell line source(s)	Human umbilical vein endothelial cells (HUVECs; primary cells) used in this study were purchased from CELL APPLICATIONS, Inc.
Authentication	Cell authentication was based on morphological criteria and expressions of the specific proteins for vascular endothelial cells (CD31 and VE-cadherin).
Mycoplasma contamination	Mycoplasma contamination of cell culture was periodically checked, and cells were found negative for the presence of mycoplasma species.
Commonly misidentified lines (See ICLAC register)	No commonly misidentified cell lines were used.

Flow Cytometry

Plots

Confirm that:

- [ ] The axis labels state the marker and fluorochrome used (e.g. CD4-FITC).
- [ ] The axis scales are clearly visible. Include numbers along axes only for bottom left plot of group (a 'group' is an analysis of identical markers).
- [ ] All plots are contour plots with outliers or pseudocolor plots.
- [x] A numerical value for number of cells or percentage (with statistics) is provided.

Methodology

Sample preparation	- Cell cycle analysis. The cells were harvested from the dish using 0.05% trypsin-EDTA, and centrifuged for 5 min at 185 × g after inactivation of the trypsin-EDTA inactivation using the cell culture medium. The collected cells were then washed with PBS and fixed in 70% ice-cold ethanol. After another PBS wash, the cell density was adjusted to 500 cells/µL. Nuclear DNA was stained using Guava Cell Cycle reagent (4500-0220, Merck Millipore) for 30 min.<br>- Cell proliferation assay. The cells were harvested from the dish using 0.05% trypsin-EDTA, and centrifuged for 5 min at 1,000 rpm after inactivation of the trypsin-EDTA with the cell culture medium. The cells were resuspended in the cell culture medium (200 µL) and stained with Guava ViaCount reagent (4000-040, Merck Millipore) for 10 min.
Instrument	Guava easyCyteTM 6HT flow cytometer (Merck Millipore)
Software	InCyte software (Merck Millipore)
Cell population abundance	No FACS sorting was performed in this study.
Gating strategy	For all experiments, debris was first excluded by a morphology gate based on FSC-H and SSC-H. All cells, stained with Guava Cell Cycle reagent (for cell cycle analysis) or Guava ViaCount reagent (for cell proliferation assay), were analyzed.

- [ ] Tick this box to confirm that a figure exemplifying the gating strategy is provided in the Supplementary Information.
